# Seasonal Changes in Patterns of Gene Expression in Avian Song Control Brain Regions

**DOI:** 10.1371/journal.pone.0035119

**Published:** 2012-04-18

**Authors:** Christopher K. Thompson, John Meitzen, Kirstin Replogle, Jenny Drnevich, Karin L. Lent, Anne Marie Wissman, Federico M. Farin, Theo K. Bammler, Richard P. Beyer, David F. Clayton, David J. Perkel, Eliot A. Brenowitz

**Affiliations:** 1 Verhaltensbiologie / Institut für Biologie, Freie Universität, Berlin, Germany; 2 Department of Neuroscience, University of Minnesota, Minneapolis, Minnesota, United States of America; 3 Department of Cell and Developmental Biology, University of Illinois Urbana-Champaign, Champaign, Illinois, United States of America; 4 Roy J. Carver Biotechnology Center, University of Illinois Urbana- Champaign, Urbana, Illinois, United States of America; 5 Department of Psychology, University of Washington, Seattle, Washington, United States of America; 6 Department of Neurobiology and Physiology, Northwestern University, Evanston, Illinois, United States of America; 7 Department of Environmental and Occupational Health Sciences, University of Washington, Seattle, Washington, United States of America; 8 Department of Otolaryngology, University of Washington, Seattle, Washington, United States of America; 9 Department of Biology, University of Washington, Seattle, Washington, United States of America; Vanderbilt University, United States of America

## Abstract

Photoperiod and hormonal cues drive dramatic seasonal changes in structure and function of the avian song control system. Little is known, however, about the patterns of gene expression associated with seasonal changes. Here we address this issue by altering the hormonal and photoperiodic conditions in seasonally-breeding Gambel's white-crowned sparrows and extracting RNA from the telencephalic song control nuclei HVC and RA across multiple time points that capture different stages of growth and regression. We chose HVC and RA because while both nuclei change in volume across seasons, the cellular mechanisms underlying these changes differ. We thus hypothesized that different genes would be expressed between HVC and RA. We tested this by using the extracted RNA to perform a cDNA microarray hybridization developed by the SoNG initiative. We then validated these results using qRT-PCR. We found that 363 genes varied by more than 1.5 fold (>log_2_ 0.585) in expression in HVC and/or RA. Supporting our hypothesis, only 59 of these 363 genes were found to vary in both nuclei, while 132 gene expression changes were HVC specific and 172 were RA specific. We then assigned many of these genes to functional categories relevant to the different mechanisms underlying seasonal change in HVC and RA, including neurogenesis, apoptosis, cell growth, dendrite arborization and axonal growth, angiogenesis, endocrinology, growth factors, and electrophysiology. This revealed categorical differences in the kinds of genes regulated in HVC and RA. These results show that different molecular programs underlie seasonal changes in HVC and RA, and that gene expression is time specific across different reproductive conditions. Our results provide insights into the complex molecular pathways that underlie adult neural plasticity.

## Introduction

The birth and death of neurons and the growth and retraction of their axonal and dendritic arbors are critical features that mediate adult plasticity of the vertebrate brain. These processes, accompanied by changes in the electrophysiological properties of neurons, permit the brain to adapt to both short term and long term environmental changes, allowing the organism to survive and successfully reproduce. One useful model system for investigating adult neural plasticity within the context of a behaviorally-relevant brain circuit is the seasonally breeding songbird.

Seasonally breeding songbirds show dramatic plasticity in adult brain and behavior between the breeding and nonbreeding seasons [1]. In these species, photoperiod-induced changes in circulating levels of steroid sex hormones induce changes in the neural structure [2], neuron number [3], and electrophysiological properties [4] of the brain regions that control behaviors related to reproduction. In songbirds, this includes song, a learned behavior used for defending territories and attracting females [5], and the song control system, a network of discrete brain nuclei that control song learning and production [6].

**Figure 1:**
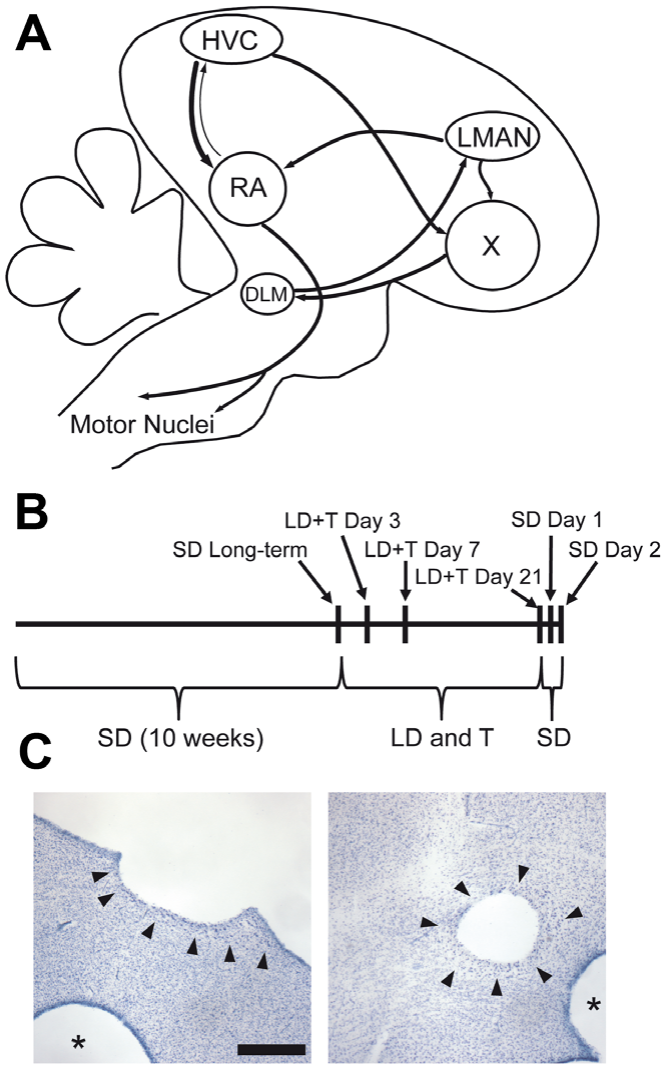
Punch location and time line for experimental methods. (A) Sagittal schematic diagram of the avian song control system. HVC (used as a proper name) projects to the robust nucleus of the arcopallium (RA), which projects to brainstem motor nuclei that regulate vocal production. The dorsal portion of RA also has a small reciprocal connection with HVC. HVC also projects to the basal ganglia nucleus Area X, which projects to the dorsolateral nucleus of the medial thalamus (DLM), which projects to the lateral magnocellular nucleus of the anterior nidopallium, which projects to RA and X. (B) Time line for the experimental procedures for this experiment. Adult male Gambel's white-crowned sparrows were held under short day photoperiod (SD) for 10 weeks, during which time they were castrated. At Day 0, one group of birds was sacrificed. The rest were transitioned to long day photoperiod (LD) and implanted with subcutaneous testosterone (T) pellets. Groups of birds were sacrificed at three time points while under LD+T (Day 3, Day 7, Day 21). On Day 21, we removed T pellets from the remaining birds, transitioned them to SD, and sacrificed them on Day 22 and Day 23. (C) Nissl images of brain sections illustrating punches of HVC (left) and RA (right). Arrowheads designate the borders of HVC and RA. Asterisks indicated punched nidopallium outside of HVC (left) and arcopallium outside of RA (right); punches outside HVC and RA were not analyzed. Scale bar = 200 µm.

Seasonal changes in song behavior and the song control system have been studied using largely morphometric and electrophysiological techniques. Little is known, however, about how breeding physiology alters gene expression within the song control system to induce changes in neural properties and ultimately song behavior, or whether the molecular mechanisms underlying seasonal changes differ across song control nuclei. Here we address these issues by analyzing patterns of gene expression in the telencephalic song nuclei HVC (used as proper name [7]), which is located in the nidopallium, and the robust nucleus of the arcopallium (RA) in white-crowned sparrows exposed to breeding or nonbreeding photoperiod and hormone conditions (Figure 1A).

While both HVC and RA seasonally grow and regress, the underlying cellular and electrophysiological mechanisms differ between the nuclei [1]. For example, HVC volume increases primarily because of the incorporation of new neurons into the nucleus, and its neurons show minimal changes in their electrophysiological properties. In contrast, RA volume increases because of changes in soma size and dendritic organization, and its neurons show substantial changes in their electrophysiological properties. These differing cellular and electrophysiological properties led us to hypothesize that different patterns of gene expression would underlie seasonal changes in HVC and RA.

To test this, we extracted RNA from HVC and RA across multiple time points designed to capture the active growth and regression of the song control system (Figure 1B). We then measured RNA expression using cDNA microarray hybridization developed by the SoNG consortium [8] and validated some of these results using qRT-PCR. Supporting our hypothesis, we found that only 16% of the genes of interest were found to seasonally vary in expression in both nuclei. We also found that the proportion of genes of interest assigned to particular functional categories differed substantially between HVC and RA. These results show that different molecular programs underlie seasonal changes in HVC and RA, and that gene expression is time specific. These results point to specific candidate mechanisms of seasonal plasticity in the song system and provide insight into the molecular complexity underlying adult neural plasticity.

## Results

In order to determine which genes vary in expression in HVC and RA across breeding conditions, we placed 36 male white-crowned sparrows (*Zonotrichia leucophrys gambelii*) under short day (SD) photoperiod to ensure they were in a photosensitive, nonbreeding state. After 10 weeks, we killed 6 males (referred to as SD long-term) and exposed the remainder to long day (LD) photoperiod and high levels of circulating testosterone (T) in order to bring them into breeding condition (LD+T). We sacrificed three groups of 6 males while under LD+T in order to capture the growth of HVC and RA (referred to as LD+T Day 3, LD+T Day 7, and LD+T Day 21, respectively). The remaining two groups of 6 males were transitioned to SD photoperiod and T was removed in order to induce regression of HVC and RA and the birds were killed (these groups are referred to as SD Day 1 and SD Day2). At sacrifice, we took punches of HVC and RA from acute brain slices. We measured mRNA extracted from the punches using cDNA microarrays developed by the SoNG initiative. The mRNA from each nucleus from each bird was hybridized on a separate array (71 arrays in total; we extracted an insufficient amount of mRNA from one HVC). We then performed statistical analyses and gene ontology (GO) analysis.

### Plasma Hormone Levels

Silastic T pellets implanted subcutaneously on day 0 increased plasma levels of T into the physiological breeding range seen in wild male sparrows under breeding conditions (Table 1) [9], [10]. After the withdrawal of T and transition to SD, levels of circulating T had dropped to basal levels when measured on SD Day 1 and Day 2 (Table 1).

**Table 1:** Plasma testosterone levels.

Treatment Group	Number of Animals	Plasma Testosterone Level (ng/ml)
Day 0, SD long-term	6	0.65±0.07
LD+T Day 3	6	10.39±2.20
LD+T Day 7	6	9.00±1.40
LD+T Day 21	6	9.81±6.23
SD Day 1	6	0.68±0.84
SD Day 2	6	0.82±0.11

Values are mean ng/mL ± S.E.M.

### Gene expression changes in HVC and RA following transitions in breeding condition relative to SD long-term

**Figure 2:**
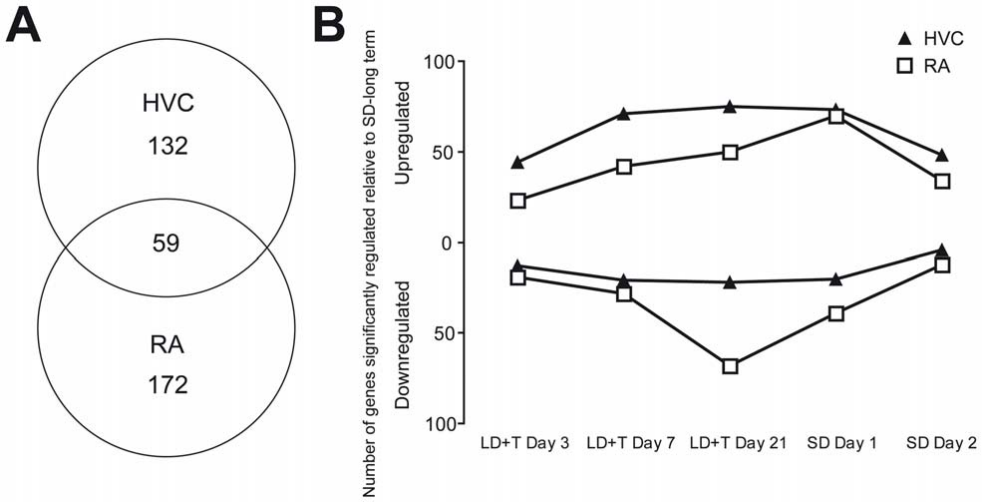
Hundreds of genes changes expression in HVC and RA with little overlap between them. (A) Venn diagram of the total number of genes that varied by more than 1.5 fold relative to the SD long-term group. Only 59 of the 363 genes were common between HVC and RA. (B) The number of genes upregulated or downregulated in HVC and RA over the different time points relative to SD-long term.

For HVC and RA, the fluorescent signals of 344 and 412 spots (excluding the control genes, see section on zenk/EGR1 below) on the array, respectively, significantly varied during at least one of the time points relative to SD long-term (Table S1 and Table S2). Spots were considered significantly varied if they changed by ≥1.5 fold in expression and had a raw p-value≤0.01. (Please see Materials and Methods for details on criteria used in determining significant change in expression and how these criteria differ in comparison to other microarray studies.) We note that the ≥1.5 fold-change (FC) criterion does not provide any additional rigor, but instead places emphasis on changes in expression that are most likely to be biologically relevant. Spots for which insufficient functional annotation was available to allow for unambiguous assignment to a gene were excluded for further analysis. Spots that were redundant for a particular gene were averaged. This left us with 191 and 231 genes in HVC and RA, respectively, that varied by more than 1.5 fold change in expression and had a raw p-value≤0.01 during at least one of the time points relative to SD long-term. We also quantified how many genes were significantly upregulated and downregulated in expression over the time course, by which we mean how many genes changed expression in at least one of the time points relative to the SD long-term group. We found that, in general, more genes were upregulated than downregulated in both HVC and RA following a transition to breeding conditions (Figure 2B). We also found that at LD+T Day 3 and LD+T Day 7 more genes were upregulated in HVC than in RA, but the number of genes downregulated was essentially the same. From LD+T Day 7 to LD+T Day 21, the number of genes upregulated was nearly the same in HVC and RA, but the number of genes downregulated in RA increased by 143%, whereas the number of genes downregulated in HVC increased only 5% at this time point. Following the transition from breeding to nonbreeding conditions, the number of genes upregulated and downregulated in HVC and RA decreased.

**Figure 3:**
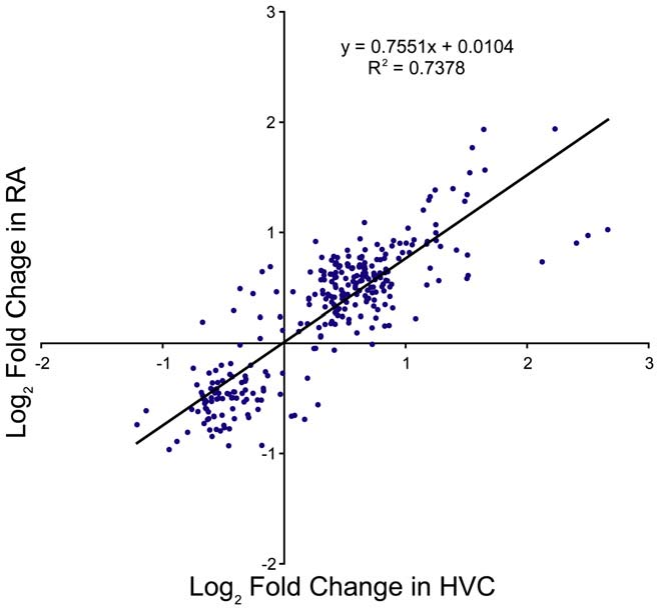
Genes in common between HVC and RA co-vary in expression. Scatter plot of the log-fold change in gene expression for the 59 genes shared between HVC and RA that varied by more than 1.5 fold in at least one time point. The expression at all five time points relative to SD-long term for each of the 59 genes is illustrated.

We found that the expression of 59 genes varied significantly in expression levels in at least one time point for both HVC and RA (Figure 2A). Expression of these genes significantly co-varied (r^2^ = 0.738, p<1×10̂-6, Figure 3). Thus, for these 59 genes that varied by ≥1.5 fold in expression and had a raw p-value≤0.01 in both HVC and RA, expression levels largely varied across both nuclei in the same direction and approximately to the same degree.

### Gene expression changes in HVC and RA following the transition from breeding to nonbreeding conditions

**Figure 4:**
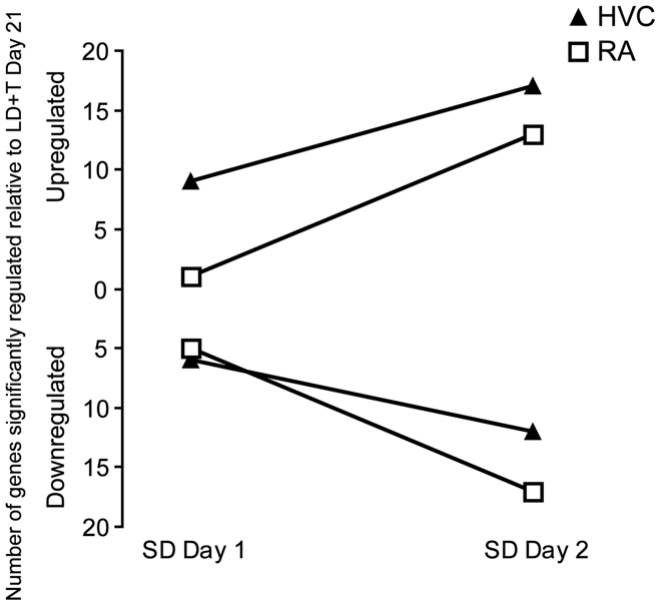
Changes in expression following the transition from breeding to nonbreeding conditions. The number of genes upregulated or downregulated in HVC and RA over SD Day 1 and SD Day 2 relative to LD+T Day 21.

Our time course includes a transition from breeding to nonbreeding conditions, which induces regression of HVC and RA [11]. In a separate analysis, we compared the expression of genes from two time points (SD Day 1 and SD Day 2) to LD+T Day 21, a time point in which both RA and HVC are fully grown [12] in order to assess how gene expression changes during this transition. In HVC and RA, 58 and 54 spots, respectively, significantly changed expression levels by more than 1.5 fold following the transition from breeding (LD+T Day 21) to nonbreeding conditions (SD Day 1 and Day 2) (Table S3 and Table S4). (Please see Materials and Methodsfor criteria details.) After accounting for redundancies and eliminating spots for which there was insufficient identification information, there were 37 and 33 genes in HVC and RA, respectively, that changed expression by more than 1.5 fold and had a p-value≤0.01 after the transition to nonbreeding conditions. For HVC and RA, 20 and 23 of these genes, respectively were not present in the data set of expression changes of genes relative to SD long-term. Almost three times as many genes were significantly regulated in HVC than in RA when expression was compared from SD Day 1 to LD+T Day 21. By SD Day 2, nearly the same number of genes was significantly regulated in RA and HVC (Figure 4).

### Gene Ontology Analysis

We submitted Ensembl IDs for all genes that significantly changed expression in HVC and RA to the GO server maintained by the Centre for Comparative and Functional Genomics at ARK-genomics (http://bioinformatics.iah.ac.uk/tools/GOfinch) that is powered by the CORNA package developed for R [13]. For HVC, five terms were found to be significantly overrepresented in our dataset, that is, there were more genes that fall into a given GO category than would be expected by chance (Table 2). Of particular note is semaphorin receptor activity and SMAD binding (see below). For RA, only “cell soma” was found to be significantly overrepresented in our dataset.

**Table 2:** Gene Ontology (GO) terms found to be significantly over-represented in HVC and RA across breeding states.

Nucleus	GO code	GO description	expected	observed	Fisher	adj.Fisher
*HVC*	GO:0017154	semaphorin receptor activity	0	4	1.00E-06	0.00068
	GO:0043025	cell soma	1	6	5.00E-05	0.017
	GO:0046332	SMAD binding	0	4	0.00022	0.035
	GO:0004574	oligo-1,6-glucosidase activity	0	2	0.00027	0.035
	GO:0004575	sucrose alpha-glucosidase activity	0	2	0.00027	0.035
*RA*	GO:0043025	cell soma	1	7	1.10E-05	0.0074

Given the relatively small number of significantly overrepresented GO terms generated by the GO analysis, we instead examined each individual gene in our dataset using publicly available databases, including PubMed (http://www.ncbi.nlm.nih.gov/pubmed/), Entrez Gene (http://www.ncbi.nlm.nih.gov/sites/entrez?db=gene), and GeneCard (http://www.genecards.org/), in order to gather more information about putative gene function. This effort revealed that gene ontology analysis, at the time of writing, does not encompass all the well-known functions for particular genes. For example, MEF2D has been officially categorized for only three GO biological process terms: transcription, regulation of transcription, and muscle development. Yet MEF2D also plays a well-characterized role in protecting neurons from apoptosis 14–22. We limited our database research only to significantly regulated spots that have been assigned an Ensembl ID and a HGNC symbol. The categorical analysis detailed below is limited to this subset of the data.

In the results that follow, we first examine genes whose expression significantly changed in nucleus HVC, and then consider those observed in nucleus RA. Given the less restrictive statistical criteria used above, we recognize that one must be cautious in focusing too much on seasonal changes of expression of individual genes because of the increased likelihood of false positives. Nevertheless, there are some interesting results in particular biological categories that are worth highlighting, including candidate genes of interest.

### HVC

**Figure 5:**
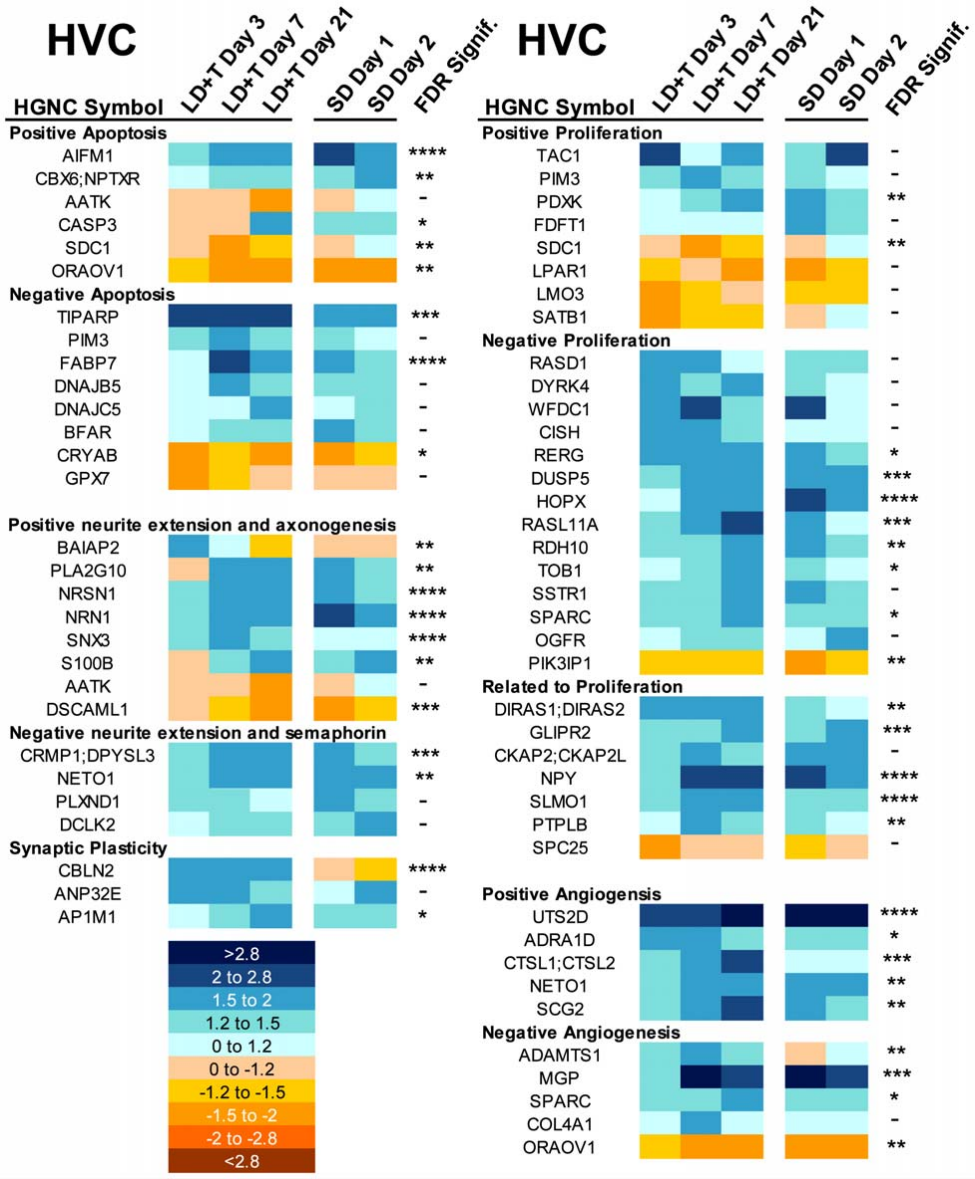
Heatmap illustrating change in expression of genes relative to SD-long term in HVC. Four broad categories are represented: Apoptosis, arborization, proliferation, and angiogenesis. A literature search was used to determine if the gene is known to enhance (positive) or inhibit (negative) a physiological function. The scale for expression is illustrated on the lower left. The more dark blue a color, the more it was upregulated relative to SD-long term. The more dark orange a color, the more it was downregulated relative to SD-long term. FDR-adjusted significance levels are also included (*>0.1, **>0.05, ***>0.01, ****>0.001).

**Apoptosis-related genes:** In general, genes that promote programmed cell death (positive apoptosis) were downregulated during exposure to breeding conditions (Figure 5). Genes known to suppress programmed cell death (negative apoptosis) were generally upregulated during exposure to breeding conditions, and many decreased in expression after transition to nonbreeding condition. The level of analysis focusing on the changes in gene expression following the transition from breeding to nonbreeding conditions revealed that eight of the 37 genes that change in HVC were related to apoptosis. One gene that increased expression after the transition to nonbreeding conditions was MEF2D, which as described above, is a well-characterized gene related to neuronal apoptosis.

**Angiogenesis-related genes:** Five genes that were classified as “positive angiogenesis.” were upregulated during exposure to breeding conditions. Likewise, many of the genes classified as “negative angiogenesis” were also upregulated during exposure to breeding conditions.

**Proliferation-related genes:** Of the eight genes that were classified as “positive proliferation”, three were upregulated and four were downregulated during exposure to breeding conditions. In contrast, of 14 genes classified as “negative proliferation” 13 were upregulated during exposure to breeding conditions. Seven other genes related to proliferation also changed expression by more than 1.5 fold over the time course.

**Arborization-related genes:** In general, genes that were classified as “positive neurite extension and axonogenesis” were upregulated during exposure to breeding conditions in at least one time point, particularly starting at LD+T Day 7 (Figure 5). Four genes were classified as “negative neurite extension and semaphorin related.” Two of these genes were upregulated during exposure to breeding conditions, and the others only increased in expression after transition from breeding conditions to non-breeding conditions. Three genes related to synaptic plasticity (CBLN2, ANP32E, AP1M1) significantly changed expression over the time course, all of which were upregulated during exposure to breeding conditions.

**Figure 6:**
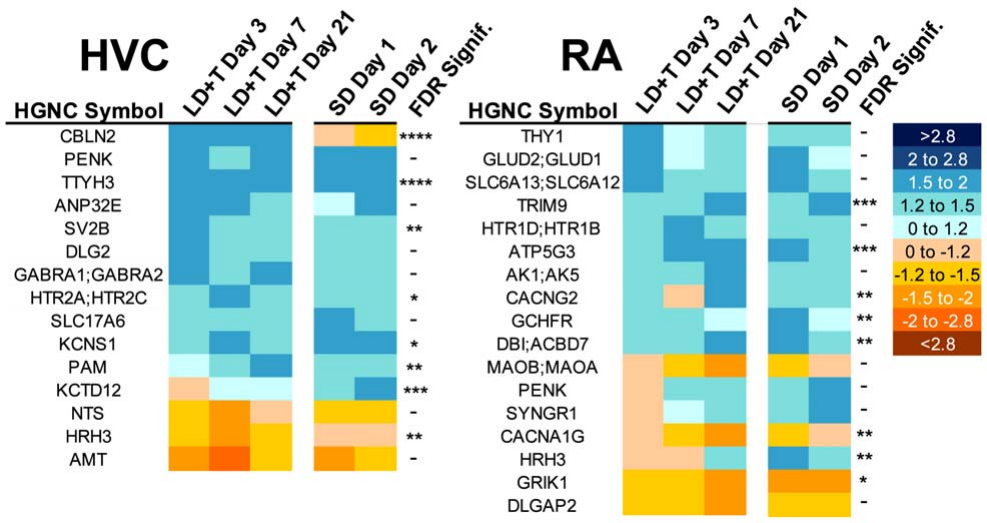
Heatmap illustrating change in expression of genes related to electrophysiology in HVC and RA. The scale for expression is illustrated on the right. FDR-adjusted significance levels are also included (*>0.1, **>0.05, ***>0.01, ****>0.001).

**Electrophysiological-related genes:** Fifteen genes related to electrophysiological properties exhibited at least one significant change in expression in HVC over the time course studied (Figure 6). These genes can be broadly divided into three different categories: those that encode ion channels, those that potentially influence synaptic properties, and those that are related to neuromodulator action. Of particular note, PENK, which encodes enkephalin, and CBLN2, which encodes the neuropeptide cerebellin, were significantly upregulated after the transition to breeding conditions, NTS, which is a precursor of the neuropeptides neuromedin N and neurotensin, was significantly downregulated after LD+T Day 7.

### HVC and RA

**Figure 7:**
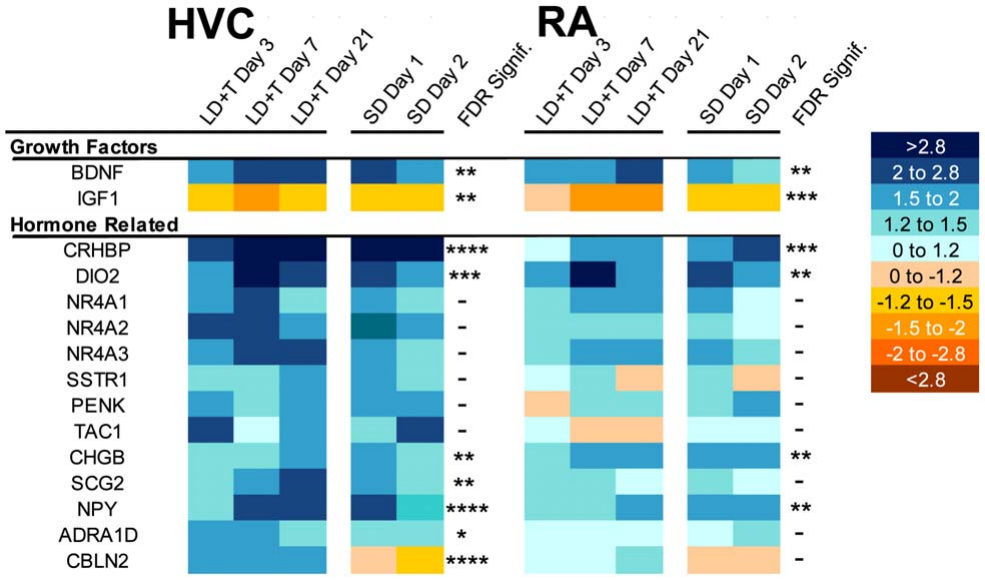
Heatmap illustrating change in expression of growth factor genes and hormone-related genes in HVC and RA. The scale for expression is illustrated on the right. FDR-adjusted significance levels are also included (*>0.1, **>0.05, ***>0.01, ****>0.001).

**Growth factors:** In HVC and RA, two growth factor genes significantly changed expression over the time course (Figure 7). BDNF was significantly upregulated after the transition to breeding conditions; IGF1 was downregulated. We also note that another trophic factor of interest, VEGF, was upregulated more than 1.5 fold in HVC at LD+T Day 7, LD+T Day 21, and SD+T Day 1, but that the p-value for these time points was greater than 0.01.

**Hormone related genes:** Thirteen hormone-related genes changed expression by more than 1.5 fold over the time course (Figure 7). In HVC, all 13 increased expression after the transition to nonbreeding conditions. The greatest change in expression detected in all of the genes for HVC was for CRHBP, an anti-corticotropin releasing factor protein, which was significantly upregulated after the transition to breeding conditions. CRHBP was also significantly regulated over the time course in RA. Three genes for nuclear hormone receptors (NR4A1-3) were significantly upregulated in HVC, and NR4A1 and NR4A3 were significantly upregulated in RA, after the transition to breeding conditions. Seven of the 13 genes upregulated in HVC were also upregulated in RA.

### RA

**Electrophysiological-related genes:** RA neurons undergo substantial changes in their intrinsic electrophysiological characteristics, which include significant increases in both spontaneous and evoked firing rates in breeding birds [23]. Genes related to electrophysiological properties that significantly changed expression in RA can broadly be divided into three categories: those that encode ion channels, those that potentially influence synaptic properties, and those that are related to neuromodulator action (Figure 6). Of particular note is GRIK1, which encodes a subunit of the kainate glutamate receptor, which is significantly downregulated in birds exposed to breeding conditions. This finding is similar to the decreased expression of the mRNA for the NMDA subunit NR2B in breeding condition canaries [24]. In addition, MAOB/MAOA, which encodes monoamine oxidase, is significantly downregulated in birds exposed to breeding conditions. This protein is necessary for inactivating the neuromodulators dopamine, noradrenaline, and serotonin.

**Figure 8:**
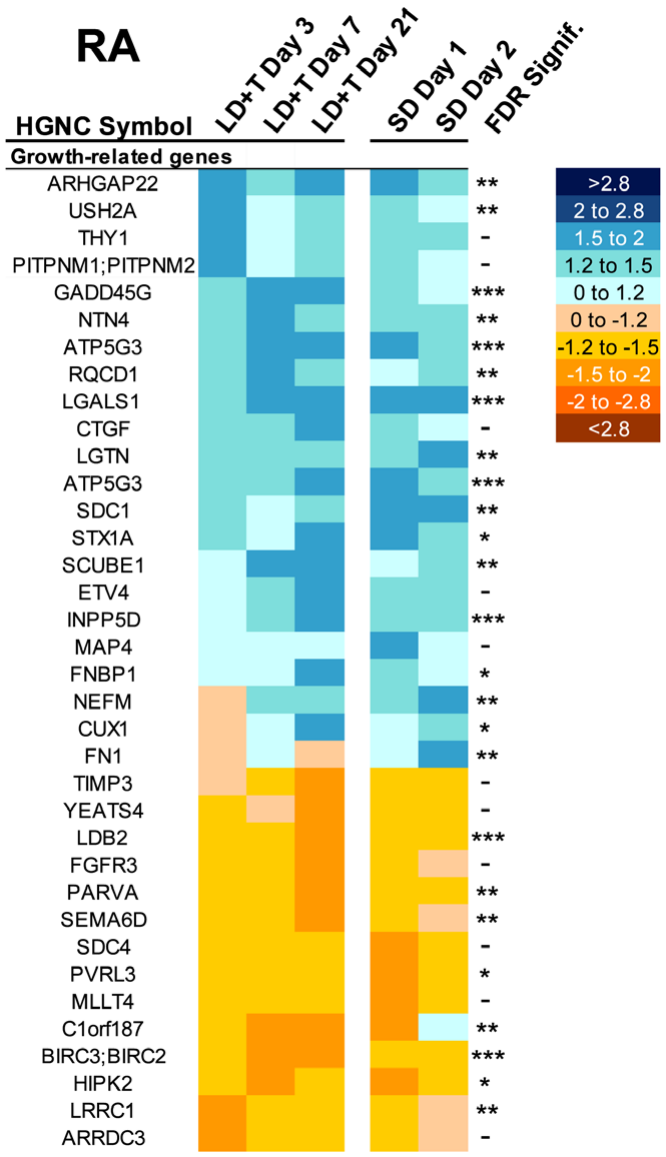
Heatmap illustrating change in expression of genes related to cell growth in RA. The scale for expression is illustrated on the right. FDR-adjusted significance levels are also included (*>0.1, **>0.05, ***>0.01, ****>0.001).

**Aborization and cell growth related genes:** We hypothesized that genes related to cell growth, aborization, and synaptogenesis would change expression by more than 1.5 fold in response to breeding conditions (Figure 8). Indeed, we found 36 genes potentially related to cell growth changed expression in RA in response to breeding conditions. Many of the upregulated genes are implicated in permitting cell growth, extracellular matrix extension, or synaptogenesis. Of the downregulated genes, the most notable were known cell growth inhibitors, including HIPK2, which encodes a co-repressor of several transcription factors.

**Figure 9:**
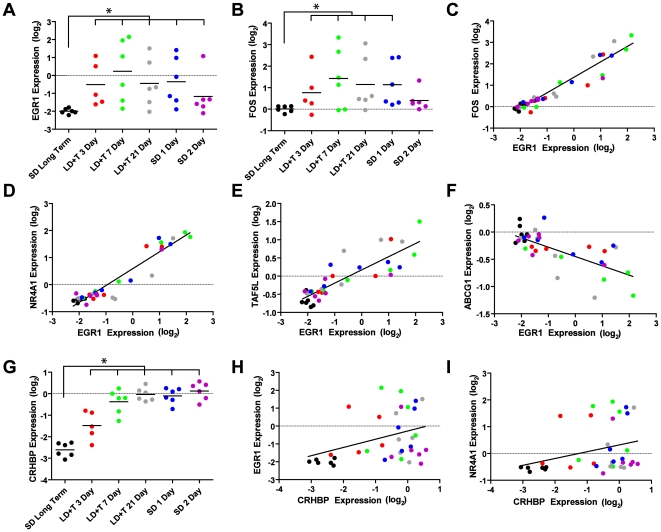
EGR1 expression correlates with expression of other genes in HVC. The expression of the immediate early genes (A) EGR1 and (B) FOS was substantially variable in each group, indicative of variable amounts of singing behavior prior to sacrifice. Despite this variability, expression of EGR1 and FOS was significantly correlated (C). Expression of EGR1 was also positively correlated with expression of a subclass of the nuclear hormone receptors, including NR4A1 (D), and many other genes, including the transcription-related gene TAF5L (E). Few genes, such as lipid-transporter ABCG1 (F), were negatively correlated with EGR1 expression. The expression of corticotropin releasing hormone binding protein (CRHBP), the gene changed the most in expression in the data set (G), did not significantly co-vary in expression with EGR1 (H) or NR4A1 (I). Expression values for all panels are illustrated relative to the universal reference sample.

**Immediate Early Genes (IEG) in HVC and RA:** The song control system is one of the leading models for understanding the relationship of IEGs to behavior [25]. Consequently, the SoNG array was designed with 48 spots for EGR1, also known as *Zenk*, which is the best studied IEG in the song control system to date [25]. We found that expression of EGR1 in both HVC and RA exhibited substantial variability in each group other than SD long-term (Figure 9A), which suggests that the rate of singing was not consistent across birds. In fact, expression of EGR1 was so variable that, though it varied by more than 1.5 fold relative to SD long-term and had a raw p-value<0.01, it had an FDR-adjusted p-value>0.1. This was also true for other IEGs, including FOS (Figure 9B).

We performed correlation analysis on EGR1 against the entire data set for HVC and RA in order to identify genes that covary with EGR1 expression, which might be considered a proxy for singing behavior right before sacrifice. In HVC and RA, we found 30 and 24 spots, respectively, that were positively correlated with EGR1 expression and had r^2^>0.5 (Table S5). Not surprisingly, known IEGs such as FOS were positively correlated with EGR1 (HVC: r^2^ = 0.905, p<0.0001, Figure 9C; RA: r^2^ = 0.835, p<0.001). Interestingly, all three members of the nerve growth factor IB-like subfamily of nuclear hormone receptor transcription factors showed striking positive correlations with EGR1, suggesting that this subfamily may also be responsive to singing behavior (e.g, HVC: NR4A1, r^2^ = 0.900, p<0.0001, Figure 9D; RA: r^2^ = 0.860, p<0.001). Other genes also correlated in expression with EGR1, including the transcription co-factor TAF5L (HVC: r^2^ = 0.724, p<0.0001, Figure 9E; RA: r^2^ = 0.684, p<0.001). In contrast to the observed positive correlations, no spots were negatively correlated with EGR1 and had r^2^>0.5, but some had r^2^ that approached 0.5 (e.g, HVC: ABCG1, r^2^ = 0.478, p<0.0001, Figure 9F).

To illustrate that correlations between EGR1 and other genes are not simply due to differences across time points and are instead due to the inherent variability of EGR1 expression in each group of animals, we compared some of the EGR1-related genes to CRHBP, the gene the varied most robustly in our data set (Figure 9G). We found the CRHBP did not substantially co-vary with the EGR1-related genes, including EGR1 itself (r^2^ = 0.135, p = 0.030, Figure 9H) and NR4A1 (r^2^ = 0.093, p = 0.074, Figure 9I).

### Categorical comparison of HVC and RA

**Figure 10:**
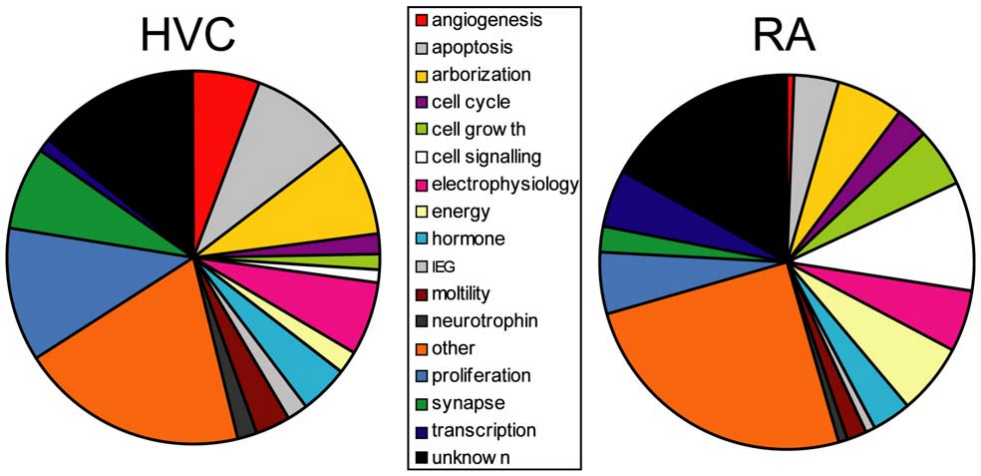
Pie charts illustrating the proportion of functional categories of genes in HVC and RA that changed expression relative to SD-long term. The proportion of genes related to angiogenesis, apoptosis, and proliferation was greater in HVC than in RA. The proportion of genes related to cell growth, cell signaling, and energy was greater in RA than in HVC.

We placed the genes in the entire data set into broad functional categories in order to compare the changes in expression in HVC and RA across the various time points. We found that there were substantial differences in the types of genes that change expression in HVC and RA (Figure 10). There was a greater proportion of genes related to angiogenesis, apoptosis, and proliferation in HVC than in RA. There was a greater proportion of genes related to cell growth, cellular signal transduction and related processes (referred to here as “cell signaling”), and energy in RA than in HVC.

### Correlation of gene expression between HVC and RA

**Figure 11:**
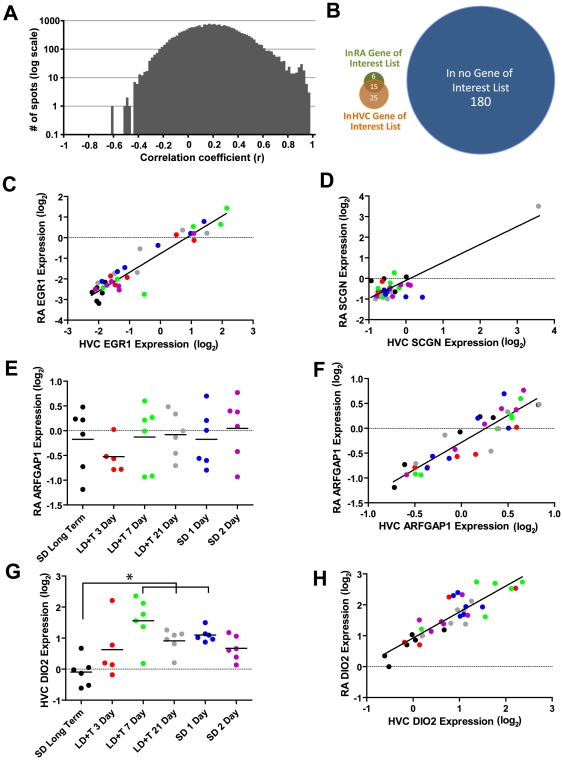
Many genes showed correlated pattern of expression between HVC and RA. (A) A frequency histogram (bin size = 0.02) of correlation coefficients for each spot on the array comparing HVC to RA is positively-skewed, indicating that genes that were correlated between HVC and RA were positively correlated. (B) Venn diagram illustrating the proportion of genes that were significantly correlated between HVC and RA that also appeared in the HVC and/or RA gene of interest lists that vary in expression across seasonal conditions. The vast majority of genes that showed correlated expression in HVC and RA were not represented in either list. (C) Expression of EGR1 in HVC was correlated with expression of EGR1 in RA. (D) SCGN is an example of a gene that had a very high Pearson's coefficient (r^2^ = 0.740) that was entirely dependent upon a single outlier. (E) Expression of ARFGAP1 did not vary significantly across breeding conditions in (F) RA or HVC but was positively correlated in expression between HVC and RA. (G) Expression of DIO2, a gene critical for thyroid hormone metabolism, varied significantly across breeding conditions in (H) HVC and RA (Table S2) and was positively correlated in expression between HVC and RA. Expression values are illustrated relative to the universal reference sample.

We performed correlation analysis comparing expression of all spots on the array between and HVC and RA for each animal in this study (n = 35, one animal was excluded due to insufficient amount of mRNA collected from the HVC punches). We found 228 spots (excluding the EGR1 array controls) that positively co-varied between HVC and RA and had r^2^>0.5. We found no spots that negatively co-varied between HVC and RA and had r^2^>0.5. Figure 11A illustrates a frequency histogram of Pearson's coefficients between HVC and RA for all spots on the array; the distribution is positively skewed (skewness = 0.24).

We compared the list of spots that co-varied between HVC and RA to the list of genes that are affected by breeding context in HVC and/or RA (Table S6). Interestingly, only 20.3% of the spots that co-varied between HVC and RA were found in our seasonal gene of interest lists (Figure 11B). EGR1 was one of the genes that significantly co-varied between HVC and RA (r^2^ = 0.891, p<0.0001, Figure 11C). Further analysis revealed that many of the “significant” correlations suffered from the fourth category from Anscombe's quartet [26], where a “strong” correlation is largely driven by the result of a single or a few outliers. One gene that followed this kind of pattern is secretagogin (SCGN, r^2^ = 0.740, p<0.0001, Figure 11D). To determine the extent of this issue, we performed outlier analysis on the distribution of gene expression for the 228 spots that positively covaried in HVC and RA. We found that 47 spots (21%) had at least one point that varied by more than three times the standard deviation of the mean in either HVC or RA. All 47 spots that had this kind of correlation followed a positive relationship. The results from outlier analysis can be found in Table S6.

ARFGAP1, which is involved in vesicle mediated transport, is an example of one gene that did not significantly vary across breeding conditions (Figure 11E) but showed a strong positive correlation in expression between HVC and RA (r^2^ = 0.830, p<0.0001, Figure 11F). DIO2, a gene that is critical for thyroid hormone synthesis, was upregulated in expression in HVC (Figure 11G) and RA (Table S2) under breeding conditions relative to SD long-term and also significantly covaried in expression between HVC and RA (r^2^ = 0.771, p<0.0001, Figure 11H).

**Figure 12:**
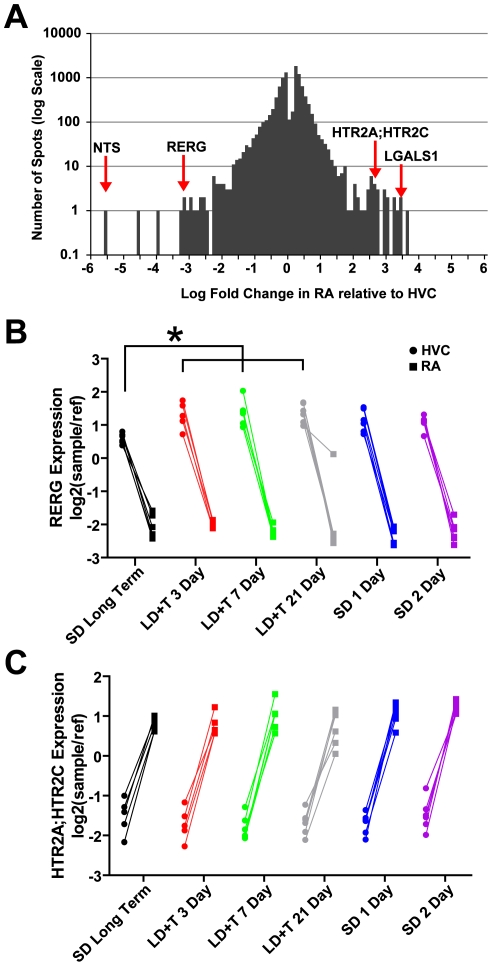
Pairwise comparisons across individual birds reveal differences in expression between HVC and RA. (A) Frequency histogram of Log_2_ expression of spots RA relative to HVC of all 9,260 spots on the array that significantly varied (p<0.05) between RA and HVC in a pairwise manner. The more negative a value, the higher the expression in HVC relative to RA; the more positive a value, the higher the expression in RA relative to HVC. Neurtensin (NTS) and Ras-like estrogen-regulated growth factor (RERG) were highly expressed in HVC relative to RA. (B) RERG expression in HVC was upregulted in HVC under breeding conditions. Expression of galectin-1 (LGALS1) and serotonin receptor 2A/2C (HTR2A;HTR2C) were significantly upregulated in RA relative to HVC. (C) Expression of HTR2A;HTR2C did not vary across breeding conditions. Expression values in B and C are illustrated relative to the universal reference sample.

We compared gene expression in HVC to RA on a bird-by-bird basis to determine if expression varied across the nuclei in a pairwise fashion. We found that 9,260 spots significantly varied between HVC and RA (Figure 12A). 80.1% and 67.9% of the spots found to significantly vary across breeding conditions in HVC and RA, respectively, were also found to significantly vary in expression across these nuclei. One example is the ras-like estrogen-regulated growth factor (RERG), which was significantly upregulated in HVC on LD+T 3 Day, 7 Day, and 21 Day relative to SD Long-Term (Figure 12B). Expression for RERG was also significantly higher in HVC in comparison to RA for all time points examined (Figure 12B). Serotonin receptors are more highly expressed in RA than in HVC; for example, expression of HTR2A/HTR2C (both are listed because the cDNA on the array is not specific enough to tell the difference between the two) does not vary across breeding conditions but is over 600% higher in RA than in HVC. Lists of spots that varied in expression in a pairwise fashion between HVC and RA with fold-change difference of expression ≥1.5 can be found in Table S7.

### qRT-PCR validation

**Figure 13:**
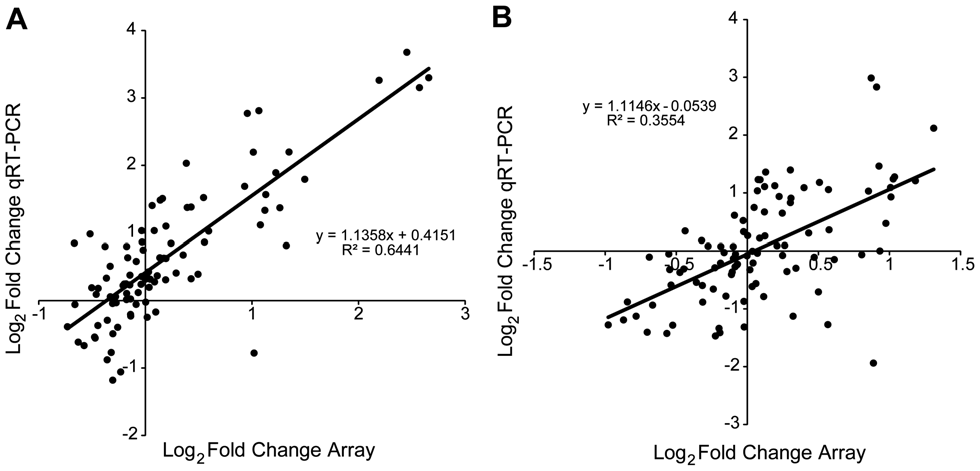
Scatter plot illustrating correlations of array results to qRT-PCR validation. The expression of 19 genes from five time points relative to SD-long term comparing results using qRT-PCR to results from the microarray are illustrated. Each point represents the group average at a given time point for a particular gene. Results assessing change in expression from the microarray were significantly correlated with results using qRT-PCR in (A) HVC (p<10^−5^) and (B) RA (p<10^−5^).

We ran qRT-PCR using RNA extracted for the arrays to validate the array results for a subset of 19 genes (BDNF, CACNA1B, CHRNA4, NOG, CRMP1;DPYSL3, FGF14, GAP43, IGF1, MT1, NTF3, VLDLR, ABCG1, AMT, COL6A3, CRHBP, MBP, NPY, NR4A3, USP35). We found that expression of genes as measured by qRT-PCR significantly correlated with expression on the array for both HVC (p<10^−5^, r^2^ = 0.644, Figure 13A) and RA (p<10^−5^, r^2^ = 0.355, Figure 13B).

## Discussion

Though seasonal plasticity of brain circuits in adult vertebrates was first described nearly 30 years ago [2], little is known about the underlying changes in gene expression. Here, we report that hundreds of genes alter expression in two song control system nuclei in the brains of adult male Gambel's white-crowned sparrows sacrificed under breeding and nonbreeding conditions. Using the SoNG-generated cDNA microarray [8], we found that 191 and 231 genes significantly changed expression by more than 1.5 fold in HVC and RA, respectively. Only 59 of these genes were shared by both nuclei, suggesting that the underlying changes in gene expression mediating seasonal plasticity in these nuclei are largely dependent upon different genetic profiles. We also characterized many of the genes that significantly alter gene expression in HVC and RA, identified categories of biological function that are known to be important contributors to brain plasticity, and examined how the pattern of expression compared between these nuclei.

The mechanisms underlying seasonal plasticity differ between HVC and RA. The increase in HVC volume in response to breeding cues is largely mediated by the recruitment of new neurons into the nucleus [1], [27]. The volumetric growth of RA, on the other hand, is primarily dependent upon increases in cell soma size and increased dendritic arborization [1]. The mechanisms underlying volumetric regression at the end of the breeding season are likewise different between HVC and RA, with HVC's regression largely mediated by programmed cell death, and RA's by cellular regression [11]. Regarding electrophysiology, the properties of HVC neurons stay largely constant across breeding conditions, while those of RA neurons dramatically change [23]. As expected from these different mechanisms, we found multiple differences in gene expression between HVC and RA, upon which our discussion will focus.

More genes were upregulated in HVC than in RA under LD+T Day 3 and Day 7 conditions. By LD+T Day 21, RA and HVC had nearly the same number of genes upregulated. The fact that more genes seem to change sooner in HVC than in RA is consistent with the differential time course of growth of these nuclei under conditions identical to those used in the current study. HVC nucleus volume reaches nearly full size after only seven days of LD+T, whereas RA nucleus volume is not fully grown until LD+T Day 21 [12]. Interestingly, the number of genes downregulated in RA increased considerably from LD+T Day 7 to LD+T Day 21, whereas the number of genes downregulated in HVC increased only a little. This differential pattern of change in gene expression may reflect that HVC has nearly completed growth by LD+T Day 7, whereas RA is still growing.

The number of genes that had significantly changed levels of expression in HVC and RA on LD+T Day 21 decreased by 48% and 59%, respectively, within two days after transition to nonbreeding conditions. This rapid reduction in the number of genes with change of expression of more than 1.5 fold is reminiscent of the rapid regression of the song control system morphometery following the transition to nonbreeding conditions [11]. We also found that three times as many genes significantly change expression in HVC than in RA just one day after the transition to nonbreeding conditions. Two days after the transition, the number of genes between the nuclei that change expression is virtually the same. This pattern of change in gene expression is reminiscent of the rapid regression of the song control system, where HVC volume is significantly regressed within 12 hours after the withdrawal of circulating T, and changes in RA morphometry are not detectable until two days later [11].

### Apoptosis related genes

Neuron number in HVC increases under breeding conditions. In the transition to nonbreeding conditions, HVC decreases in volume and neuron number within four days [11]. This process is known to be caspase-dependent [28], which strongly suggests a role for programmed cell death. We found that 14 genes related to apoptosis altered their expression in response to breeding conditions. Genes known to promote programmed cell death generally were downregulated under LD+T and upregulated after the transition to SD, consistent with previous results [11], [28]. Also consistent with apoptotic processes occurring in HVC is the fact that SYNGAP1 was upregulated only on SD Day 2. This gene is known to be upregulated in cells undergoing apoptosis, though its exact role is unknown [29].

One exception to the overall pattern of apoptosis-related genes, though, was caspase-3 (CASP3), an important mediator of apoptosis. CASP3 showed increased expression at LD+T Day 21 relative to SD. This may not be functionally significant, though, because CASP3 is known to be present in an inactive form in significant quantities in all cells and must be activated by phosphorylation in order to carry out programmed cell death. This process is largely independent of changes in mRNA levels. Thus, this increase may not reflect an increase in ongoing apoptosis but instead indicate an increase in stores of CASP3, and may even play an alternative role in synaptic regulation, a process already described in the songbird telencephalon [30].

Another gene that changed expression by more than 1.5 fold across the time course was S100B, a neurotrophin that plays a number of important roles related to plasticity. It was upregulated on LD+T Day 21 and SD Day 2. Its role related to apoptosis is complicated; relatively low levels of expression promote survival, but high levels of expression promote cell death [31]. S100B may play a similar role in HVC, because it is specifically expressed in HVC relative to surround [32]

### Angiogenesis related genes

As HVC grows, there is an increase in its vasculature, which is influenced by the action of VEGF [33]. We found 10 genes related to angiogenesis that changed expression by more than 1.5 fold in at least one of the five time points relative to SD-long term. Five of these are known to promote angiogenesis, and all five were upregulated under LD+T, when the vasculature in HVC would be growing. The other five genes are anti-angiogenic and also had elevated expression under LD+T, except for ORAOV1, which was downregulated and is known to have a multitude of effects on cell growth as well as angiogenesis [34]. The upregulation of anti-angiogenic genes may be a result of a homeostatic-like process, ensuring that the pro-angiogenesis processes are kept in check. Consistent with this hypothesis is the fact that the anti-angiogenic genes are not upregulated until LD+T Day 7, whereas UTS2D and ADRA1D, two genes known to promote angiogenesis [35], [36], are upregulated by LD+T Day 3. In addition we found that the expression of VEGF increased by more than 1.5 fold under breeding conditions, but this change was not statistically significant in our analysis. This is particularly interesting given the role VEGF plays in angiogenesis in HVC [33], [37], [38].

### Proliferation related genes

HVC increases in volume and neuron number after transition to breeding conditions. Despite this, the rate of neuronal recruitment is actually *lower* in birds sacrificed under breeding conditions than under nonbreeding conditions [39], [40]. The pattern of expression we found in the 28 genes related to cellular proliferation that changed in expression levels in HVC in at least one of the five time points relative to SD long term is consistent with the lower rate of recruitment observed under breeding conditions. Fourteen were classified as negative proliferation, and 13 of those were upregulated under LD+T. Likewise, four of the eight genes that we classified as positive for proliferation were downregulated under LD+T. One gene in particular, TAC1, showed a very unusual expression pattern, being highly upregulated only on LD+T Day 3 and SD Day 2. TAC1, also known as Substance P, is known to promote proliferation under normal and ischemic conditions [41]. TAC1 may be important not only in increasing HVC neuron number, which occurs in the days immediately following the transition to LD+T, but also when HVC is regressing; neuron loss is necessary for the subsequent increase in the rate of neuronal recruitment [42].

### Arborization and cell growth related genes

Seasonal growth of HVC involves the recruitment of new neurons into an existing circuit. Newly recruited neurons require dendritic and axonal arborization. We found that 15 genes related to arborization altered their expression in at least one of the five time points relative to SD-long term. In general, genes that promote neurite extension were upregulated at LD+T Day 7, and other genes that promote neurtie extension were down regulated at LD+T Day 21. This pattern is consistent with the result that HVC is largely done growing by LD+T Day 7 [12]. Several other genes that negatively-regulate neurite extension, including semaphorin receptor genes, showed a mixed pattern of expression over the time course. When we compared expression of genes after the transition to nonbreeding conditions using LD+T Day 21 as a baseline, however, we found that three semaphorin receptor genes (PLXND1, PLXNA1, PLXNA4) increased expression. These changes may in part mediate the rapid changes in HVC neuron density observed after the transition to nonbreeding conditions. Nevertheless, the overall mixed pattern of arborization-related gene expression may be expected given that neurite extension is a homeostatic process.

RA neuron soma size [1], dendrite length [43], and spine number [44] also increase in response to breeding conditions. These changes are accompanied by an increased number of axonal projections from HVC neurons into RA [40], [45]–[47], which presumably increases the number of synapses made onto RA neurons. As predicted from this previous research, the current study found that expression of several genes related to cell growth and synaptogenesis changed in LD+T birds. The time course of the changes in gene expression is consistent with that of RA cell growth. Six genes were changed by LD+T Day 3, nine genes by LD+T Day 7, and 20 genes by LD+T Day 21. RA soma size reaches its maximum between seven and 21 days after initial exposure to breeding conditions [12], [48]. The time course of dendritic arbor expansion is unknown, although some insight can be gleaned from the time course of the growth of RA nucleus volume, which reaches a maximum between seven and 21 days after initial exposure to breeding conditions [12].

### Growth factors

Two growth factors varied by more than 1.5 fold in expression over the time course (Figure 7). BDNF was upregulated after the transition to breeding conditions and is known to be an important mediator of plasticity in the song control system. BDNF regulates neurogenesis in HVC [49], is regulated by sex steroids [50] and amount of singing [51], and may contribute to sexual dimorphism of the song control system [52]. In white-crowned sparrows, BDNF gates seasonal change of RA soma size and neuron density [53]. IGF1 was downregulated in HVC and RA while under breeding conditions. In HVC, IGF1 is known to promote neuronal migration and differentiation [54]. Thus, the fact that IGF1 is downregulated is consistent with the anti-proliferative state of HVC while under breeding conditions.

### Hormone related genes

Hormones regulate the seasonal plasticity of song control system morphology and physiology. We found 13 genes related to hormones that varied in expression by more than 1.5 fold in at least one of the five time points relative to SD long-term (Figure 7). All 13 were significantly upregulated in HVC after the transition to nonbreeding conditions; seven of the 13 were significantly upregulated in RA over the time course. Xie et al [55] analyzed the same array used in this study and identified 31 prohormone genes represented by 41 spots. 22.6% of the genes they identified are also upregulated in HVC in our study. Most of the research on hormone-dependent seasonal plasticity of the song control system has focused on sex steroids. Our results indicate a number of other hormones and neuropeptides may also play an important role in regulating seasonal plasticity of the song control system, including corticosterone, thyroid hormone, somatostatin, enkephalin, tachykinin peptide hormones, secretogranin, neuropeptide Y, catecholamines, and cerebellin. Substance P, a tachykinin peptide hormone, enkephalin, and somatostatin have previously been shown to be expressed in HVC of zebra finches (*Taeniopygia guttata*) [56], [57]. The expression of several genes related to thyroid hormone regulation varies seasonally in the hypothalamus of song sparrows [58]. In addition, recent work has shown that corticosterone treatment induces regression of HVC in song sparrow [59] and reduces rate of proliferation in the ventricular zone in male zebra finches [60]. This is especially interesting given that our data set showed that CRHBP, an anti-corticotropin releasing binding protein, was significantly upregulated after the transition to breeding conditions. Indeed, seasonal changes in CRHBP expression were greater than any other gene in our data set. CRHBP in HVC and RA may prevent the action of CRH-like peptides or may play a functional role itself in a variety of cellular processes [61]. Future work should investigate the contribution of these other hormones to seasonal regulation of the song control system.

### Electrophysiological related genes

A number of genes that could potentially impact the electrophysiological properties of HVC neurons showed changes in expression greater than 1.5 fold in response to breeding conditions. For example, TTYH3, which encodes a calcium-activated large conductance chloride channel, was upregulated three days after exposure to breeding conditions and remained upregulated throughout the rest of the LD+T time points. Increased expression of this channel could allow more robust calcium-triggered hyperpolarization of HVC neurons. Other genes of interest in HVC relate to neuromodulators. CBLN2, which encodes a neuropeptide implicated in cerebellar long-term depression, was upregulated in HVC beginning three days after initial exposure to breeding conditions, and down regulated under non-breeding conditions. A serotonin receptor (HTR2, the cDNA spots on the array cannot distinguish the subtype) was upregulated, consistent with reports of increased sensitivity to serotonin in breeding tree-sparrows [62] and the role of serotonin in mediating aggression in song sparrows [63].

The electrophysiological properties of RA neurons change upon exposure to breeding conditions, with significant increases in spontaneous and evoked firing rate [4], [23], [48], [64], membrane time constant [23], and cellular capacitance [23]. The ionic or second messenger systems underlying these changes are unknown. We were thus particularly interested in genes whose products could influence RA neuron electrophysiological properties. An interesting observation from our analysis is the downregulation of CACNA1G, which encodes the T-type calcium channel subunit cav 3.1. T-type calcium channels are known regulators of intrinsic neural spiking and pacemaker activity [65], consistent with the changes in RA neuron spontaneous firing rate.

### Seasonal regulation of HVC-specific genes

A previously published array study showed that there are many genes that are differentially expressed between HVC and HVC shelf, the region of nidopallium just ventral to HVC in adult male zebra finches [32]. We found that 7.1% of the genes they identified as being HVC-specific are also found in our HVC data set of seasonal regulated genes. In addition, some of the genes that overlap between these two studies (e.g. CRHBP and S100B) showed some of the largest changes in expression in our data set. This substantial overlap between these two studies is not entirely surprising, given that the robust seasonal changes that occur in HVC likely do not happen in the surrounding tissue, including HVC shelf. Thus it would be expected that some of the genes that are differentially expressed between HVC and HVC shelf would also contribute to, or at the very least alter expression during seasonal changes in HVC morphology. We predict that at least some of the 7.1% of the genes overlapping between our study and that of Lovell et al. [32] may be regulated across development in zebra finches, which is influenced at least in part by the action of sex steroid hormones [66].

A well-characterized example of behavioral regulation of genetic expression is singing-induced upregulation of EGR1 (also known as ZENK) in song control system nuclei. In male zebra finches, expression of EGR1 mRNA is highest 30 minutes following a bout of singing and decreases to almost undetectable levels after another 30 minutes of silence [67]. Male white-crowned sparrows under laboratory conditions sing at very high rates while under breeding conditions and rarely under nonbreeding conditions [68]. Nevertheless, we did not record individual animals because they were kept in group housing and song rate for individual birds can vary enormously from day to day [68]. We therefore cannot directly compare song rate and expression of IEGs such as EGR1 within individual birds. That said, we did find that EGR1 was on average expressed at higher levels under breeding than nonbreeding conditions (Figure 9A). In addition, we found dozens of genes that significantly co-varied with EGR1 expression in HVC and RA. The fact that EGR1 expression was strongly correlated with expression of a number of known IEGs, including FOS and ARC [69], [70], suggests that song activity prior to sacrifice may have increased IEG expression in some animals, which might explain why EGR1 expression had the third greatest inter-animal variability out of 20,000 spots on the array. We also found that EGR1 expression was strongly correlated with all three members of the nerve growth factor IB-like subfamily of nuclear receptor transcription factors, suggesting that this expression family may be driven by singing. This subfamily is known to be responsive to a diverse range of stimuli and may play a role in energy homeostasis and steroid hormone signalling [71]. We must note, however, that correlations are not themselves conclusive, and future experiments in white-crowned sparrows that include careful analysis of individual song behavior prior to sacrifice is needed.

### Comparison of changes in HVC and RA

A comparison of categories of the genes that changed expression seasonally in HVC and RA revealed important differences (Figure 10). The proportion of apoptosis-related and proliferation-related genes was much greater in HVC than in RA. This is consistent with the different mechanisms of seasonal change between HVC and RA: seasonal changes in HVC are driven by changes in neuron number, whereas neuron number in RA stays constant across breeding conditions [72]. There was a greater proportion of genes dedicated to angiogenesis in HVC than in RA as well. Hormonally-driven changes in HVC angiogenesis and its contributions to changes in neuronal recruitment are well-documented [33], [37], [38], [73]. The fact that many fewer angiogenesis-related genes change expression in RA suggests that this region does not undergo the kind of vascular changes seen in HVC.

We found that a greater proportion of genes related to cell growth changed expression in RA than in HVC. This is expected given that neurons in RA undergo substantial changes in soma area across breeding conditions, whereas changes in HVC soma area are not as great and in some studies not even reach significance. The fact that a greater proportion of genes that changed expression in RA is related to cell signaling and energy use may be due to several factors. The spontaneous firing rate of RA neurons various across seasons, which probably is related to changes in cell signaling. These changes in electrical activity drive changes in energy use. Another factor may be methodological. RA is largely made up of neurons that project to the brainstem, in addition to a smaller population of interneurons. HVC, on the other hand, in addition to a heterogeneous population of interneurons, is largely made up of two sets of projection neurons, and only one (at least in zebra finches) undergoes neuronal turnover in adults [46]. It may be that the different types of neurons in HVC undergo substantial changes in cell signaling and energy use, but pooling them all into one tissue punch of the whole nucleus obscures any differences. Likewise, our punches generally included the proliferative ventricular zone just dorsal to HVC (Figure 1C), which can further obscure or alter changes in gene expression in our data set. Laser capture microdissection of particular neuron types in HVC would unravel this question; such techniques have found differential expression of genes between the two populations of HVC projection neurons in adult male zebra finches [74].

We also examined whether expression in HVC and RA is correlated across animals. We found that 275 spots were positively correlated between HVC and RA, whereas none of more than 20,000 spots on the array were negatively correlated. Though the genes that change in expression depending upon breeding context do not show much overlap between HVC and RA (Figure 11B), these correlations indicate that genes exclusively positively co-vary in expression. Of course, these correlations may not be restricted to just HVC and RA. Gene expression could easily co-vary between the nidopallium, where HVC resides, and the arcopallium, where RA resides. Thus these correlations could be the result of coordinated gene expression patterns across the telencephalon or even the whole brain. It is necessary to compare gene expression in HVC and RA to areas surrounding these nuclei in order to determine whether these patterns are song system specific. Nevertheless, it is clear that if gene expression in HVC and RA co-varies, it does so positively.

We found that expression for 9,260 spots on the array, nearly half of all spots, significantly varied between RA and HVC. Of these differences, however, 86% were less than 1.5 fold, which suggests that the majority of these differences, though statistically significant, may be of limited biological significance (though we note that small changes in expression of certain genes, such as transcription factors, can have substantial biological effects). Similarly, the differences detected here may not be limited to just RA and HVC but also may include surrounding arcopallium and nidopallium. Nevertheless, 1,306 spots significantly varied between HVC and RA by more than 1.5 fold in expression. Some examples include neurotensin (NTS), which appeared to be expressed almost 50 times more in HVC than in RA (Figure 12A). NTS is neuropeptide that may play a role in modulating dopamine signaling. Another gene that was highly expressed in HVC relative to RA was ras-like estrogen-regulated growth factor (RERG), which lowers the proliferation rate of dividing cells [75]. Serotonin-receptors were significantly upregulated in RA relative to HVC. This is particularly interesting because serotonin was recently shown to enhance endogenous firing of RA neurons via activation of the HTR2 subunit [76]. Expression of galectin-1 (LGALS1) was also upregulated in RA relative to HVC; galectin-1 may play a number of roles in the brain, including regulation of proliferation rate and assisting in recovery from neuronal injury [77].

Here we present the first comprehensive analysis of gene expression in the song control system in response to changing breeding conditions. We found significant differences in gene expression patterns between HVC and RA during seasonal growth and regression, which is consistent with the differing cellular and electrophysiological profiles of these nuclei. We conclude that gene expression shows region and time specific changes associated with the different mechanisms underlying seasonal plasticity.

## Materials and Methods

### Ethics Statement

The Institutional Animal Care and Use Committee at the University of Washington approved all procedures used in this study, listed in detail below (permit # 2008-06). The birds used in this study were collected under State of WA Scientific Collecting permit # 10-162 and U.S. Fish and Wildlife Permit #MB708576-0.

### Animals

We collected 36 adult male Gambel's white-crowned sparrows in eastern Washington during their autumnal migration. We housed the birds in outdoor aviaries for up to 30 weeks prior to placing them in indoor aviaries. Once indoors, they were exposed to a short-day photoperiod (SD; 8 h light: 16 h dark) for at least 10 weeks before use to ensure that they were photosensitive and, therefore, responsive to circulating plasma T and the long day photoperiod typical of their Alaskan breeding grounds (LD: 20 h light: 4 h dark). Birds kept on SD maintain regressed testes and song nuclei, basal levels of T typical of the non-breeding season, low song rates and unstereotyped song structure, and a low intrinsic spontaneous firing rate in the robust nucleus of the arcopallium (RA; a pre-motor song control nucleus) [1], [4], [10], [12], [64]. Food and water were available ad libitum throughout the experiment. Birds were group housed in indoor cages and could see and hear the other birds housed in the same room. We recorded birds prior to sacrifice, but given that they were housed in groups, it was impossible to measure song rate of individuals prior to sacrifice. We castrated all birds during this initial SD exposure to eliminate the presence of gonadal hormones. To castrate birds we anesthetized them with isoflurane, made a small incision on the left side anterior to the caudal-most rib and dorsal to the uncinate process, and aspirated the testes. This procedure was completed within 10 min. We confirmed that castration was successful at sacrifice.

### Systemic hormone and photoperiod manipulations

After the initial 10 weeks of SD photoperiod exposure, we implanted birds with a single subcutaneous Silastic capsule of T, and shifted them to LD photoperiod for 21 days. This period is sufficient to induce full growth of the song nuclei [12] neuronal electrophysiological properties typical of the breeding season [4], [48], and maximally increase song stereotypy and rate [10], [78]. We made the capsules from Silastic tubing (i.d. 1.0 mm; o.d. 2.0 mm; length: 12 mm; VWR, West Chester, PA) filled with crystalline T (∼0.017 g) as in Tramontin et al., [79], rinsed them with ethanol, and soaked them overnight in 0.1 M phosphate-buffered saline (PBS) prior to implantation. We implanted birds housed on LD with T capsules because exposure of wild-caught birds to LD alone in the laboratory does not elevate circulating T levels into the physiological breeding range observed in wild Gambel's white-crowned [9], [10], and to reduce the variability of circulating T levels across individual birds. We note that circulating steroid hormone levels are not necessarily identical to those in the brain parenchyma, due to local neurosteroid synthesis [80].

On day 21 we removed the subcutaneous T capsules of the birds, and shifted them back onto SD photoperiod for 10 more weeks. With their cycles thus synchronized, on Day 0 we sacrificed 6 birds. This group of birds, referred to as SD long-term, represents the steady-state of the regressed song control system and served as a baseline for all other groups. We implanted the rest of the birds with a subcutaneous Silastic capsule of T, and shifted them to LD photoperiod (Figure 1B). On Days 3, 7, and 21 of exposure we sacrificed 6 birds (LD+T Day 3, LD+T Day 7, and LD+T Day 21, respectively). We chose to sacrifice birds on day 3 and day 7 to measure gene expression while the song control system was actively changing from a nonbreeding to a breeding phenotype. By day 21, the song control system has reached its full breeding phenotype [12], and this time point represents the steady state of the breeding-state song control system. On day 21, we removed the subcutaneous T capsules from the remaining birds and shifted them overnight to SD photoperiod to induce regression of the song control system back to the non-breeding state [11]. On Days 22 and 23 we sacrificed 6 birds (SD Day 1 and SD Day 2, respectively) to measure gene expression while the song control system is actively regressing. To control for circadian patterns of gene expression, all birds were sacrificed during the three hour period following their subjective dawn (i.e., lights on).

We started sacrificing birds two hours after lights-on at a pace of one animal per hour with three birds killed per day. At sacrifice, each animal was anesthetized with isoflurane and killed by decapitation. The brain was dissected rapidly into ice-cold, oxygenated artificial cerebral spinal fluid (ACSF) containing the following (in mM): 119 NaCl, 2.5 KCl, 1.3 MgSO4, 2.5 CaCl2, 1 NaH2PO4, 16.2 NaHCO3, 11 D-glucose, and 10 HEPES, osmolarity adjusted to 350 mOsm with sucrose. Parasagittal brain slices (300 µm thick) were prepared using a Vibratome (Vibratome). From these slices, we collected punches of tissue containing only HVC and RA from both hemispheres (verified post-hoc by Nissl-staining of re-sectioned, fixed tissue, as in [48]). We should note that HVC punches generally included the proliferative ventricular zone just dorsal to HVC, which could alter the results obtained in this study. The tissue punches were flash frozen in a dry ice/ethanol slurry, and stored at −80 degrees C until processed for microarray hybridization.

### RNA isolation and microarray hybridization

Following the standard procedures of the SoNG Initiative [8], tissues were sent to the Keck Center for Comparative Genomics at the University of Illinois, where mRNA extraction and hybridization was performed [8]. Briefly, total RNA was extracted (RNaqueous Micro, Ambion, Austin, TX), DNAase I treated, double-amplified (MessageAmp II aRNA Kit, Ambion, Austin, TX), and Cy3/Cy5 dye-coupled (GE Life Sciences, Piscataway, NJ) as previously described [8]. RNA from individual animals (n = 6) were hybridized individually within each time point. To enable cross-batch normalizations and analysis of the tissue, each array was hybridized with one of the experimental samples and a universal reference sample. The universal reference sample was a pooled composite of zebra finch brain mRNA that was hybridized on all of the SoNG microarrays [8]. The Cy3/Cy5 dye coupling was balanced (“dye-flipped”) between experimental and universal reference samples within each treatment group to control for potential dye incorporation and hybridization biases.

The labeled samples were hybridized to the SoNG 20 K microarray designed for use in the zebra finch, the composition of which is described in detail in Replogle et al. [8]. Briefly, the array contains spotted cDNAs representing 17,214 different transcripts that are nonredundant in that each one contains at least some unique sequence relative to all others on the array. Mapping of these against the SoNG EST collection suggests that they represent the products of 11,500–15,000 genes. Redundancies represent either non-overlapping stretches of the same transcript or alternative splice forms. The array also contains 368 known control sequences including EGR1 and *β-actin*.

The arrays were hybridized overnight at 42 degrees C, washed, scanned (Axon GenePix 4000B, Molecular Devices, Sunnyvale, CA), and visualized with GenePix Pro 6.0 (Molecular Devices) as described for all SoNG arrays [8]. Additional information is publicly accessible via internet at the Songbird Neurogenomics website (http://titan.biotec.uiuc.edu/songbird/), which includes a link to the ESTIMA:Songbird EST database (http://www.uiuc.edu/goto/songbird). All ESTs have been deposited in Genbank (Accession numbers DV944971–DV962014, CK301200–CK317559 and FE712085–FE739917). Raw microarray data is archived and distributed in MIAME-compliant form using NIH's GEO (Gene Expression Omnibus) repository. (Accession number GSE28347)

### Microarray statistical analysis

Data normalization and statistical analysis of the microarray data were carried out with R statistical software packages from BioConductor (http://www.bioconductor.org/). The mean foreground values for all spots were used without background correction. Within-array normalization was done using the “printtiploess” method and between-array normalization was done using the “Aquantile” method. No spots were filtered out prior to statistical analysis. Within- and between group comparisons were calculated using the limma package in Bioconductor which uses a modified t-test to calculate p-values using an empirical Bayesian method to moderate the standard errors of the estimated log-fold changes [81]. Limma uses variance information from all the spots on the array to arrive at an estimate of per spot variance used in the t-tests. p-values were adjusted for multiple comparisons with the program q-value [82], which allows for selecting statistically significant genes while controlling the estimated “false discovery rate” (FDR). Because of inherent variability in our results due to cross-species hybridization issues, we felt that relying on FDR-adjusted p-values was too restrictive. We therefore performed the alternative analysis described below. Nevertheless, we have included FDR-adjusted significance values in our tables and heatmaps so that readers can better judge the data for themselves.

We used a balanced approach in setting criteria for selection genes. First, we set the alpha level to 0.01 and used raw p-values (not statistically adjusted for multiple comparisons, see above) to determine if genes significantly varied in expression. This relatively relaxed statistical cutoff is necessary in order to account for the fact that cross-species hybridization introduces additional variability into the array results that can result in false negatives. In fact, this criterion was used in another cross-species study using this same microarray [58]. Then, based on previously published microarray methodology [83], we set a threshold of 1.5 fold change in expression levels above or below the baseline in order to identify the most biologically-relevant changes. Thus our data set was restricted to those genes that had raw p-values<0.01 and showed a fold change of at least 1.5 in at least one time point relative to SD long-term or for the transition to nonbreeding conditions relative to LD+T Day 21. We considered any spot that met these criteria to have significantly varied in expression. We recognize, however, that these criteria are more permissive than more widely-accepted statistical standards for microarray analysis and that our data set likely includes some false positives. To help the reader make a better assessment of statistical differences, we also included the FDR-adjusted p-values in the heatmaps and tables. At each time point we collected the SoNG ID number for each spot that met these criteria and determined the expression for all significantly-changed genes across all time points. We determined the Ensembl IDs and HGNC symbols for the cDNAs on the array using the mapping provided in the supplement to Warren et al. [84]. This procedure revealed that many genes on the array were redundant; that is, a given gene was represented by more than one spot. We therefore averaged the fold change in expression levels across redundant spots for each gene.

There were two types of transition in our timeline: nonbreeding->breeding (SD long-term to LD+T time points) and breeding->nonbreeding (LD+T Day 21 to SD time points). To take into account these two transitions, we used two types of comparison to assess changes in gene expression. First, we assessed average expression levels of each spot (defined as expression of mRNA from an experimental bird relative to the zebra finch reference pool) for birds sacrificed at LD+T Day 3, LD+T Day 7, LD+T Day 21, SD Day 1, and SD Day 2 and compared them to average expression levels for each spot from birds sacrificed on SD long-term. Thus, all changes in expression were calculated as fold change of expression levels using SD long-term as the baseline for the other five time points. We used the comparison to SD long-term as the baseline for the majority of our analysis because SD long-term is arguably the time point in which HVC and RA are most likely to be stable (i.e. not undergoing seasonal change). The second type takes into account the transition from breeding to nonbreeding conditions, which induces regression of song control system nuclei, including HVC and RA [11]. We compared the expression levels of genes from two time points (SD Day 1 and SD Day 2) to LD+T Day 21, a time point in which both RA and HVC are fully grown [12] in order to assess how gene expression changes during this transition. This second type takes into account the fact that some genes may significantly change expression levels relative to LD+T Day 21 but not relative to SD long-term. In other words, expression of a gene may fluctuate in expression by less than 1.5 fold relative to SD long-term over the time course but may significantly change expression by more than 1.5 fold relative to LD+T Day 21.

To identify genes that co-varied or differed in expression between HVC and RA, we compared expression levels of HVC to RA of each spot on the array for 35 animals in the study (one animal had to be excluded because of insufficient mRNA extracted from HVC punches). Because half the arrays had the common reference in the R channel and half had it in the G channel, instead of using log2(R/G) values for the correlation analysis we converted them all to log2(sample/reference). We limited our list of significant correlations between HVC and RA to those spots that had and r^2^>0.5 and p-value<0.05. For correlations between HVC and RA, we performed outlier analysis on this list to account for “significant” correlations that were largely driven by the expression levels of genes from one or a few animals. The outlier analysis consisted of determining if the expression level of any animal in either HVC and/or RA varied >3 times the standard deviation surrounding the group mean. To identify differences in expression between HVC and RA, we performed paired t-tests.

### Categorical Analysis of HVC and RA genes of interest

Given the limited utility of GO analysis (see Results), we undertook an effort to categorize all 363 genes that varied in expression across breeding conditions relative the SD long-term time point in HVC and RA using a thorough database search. We examined each gene in our dataset using publicly available databases, including PubMed (http://www.ncbi.nlm.nih.gov/pubmed/), Entrez Gene (http://www.ncbi.nlm.nih.gov/sites/entrez?db=gene), and GeneCard (http://www.genecards.org/), in order to gather as much information as possible about putative gene function. We placed the genes into broad functional categories in order to compare the changes in expression in HVC and RA across the various time points. The proportion of genes falling into a calculated was divided by the total number of genes for HVC and RA. Genes for which insufficient information could be gathered were placed into an “unknown” category. Genes that had some function not related to the ones listed were placed into an “other” category. We used the preponderance of evidence in order to assign genes to only a single category. We recognize, though, that our list includes genes that have more than one function, and that this is one potential shortcoming of this methodology. The differences between HVC and RA in the proportion of genes falling into different categories were not statistically analyzed.

### Sequence verification of Gambel's white-crowned sparrow cDNA sequences

cDNA sequence information for Gambel's white-crowned sparrow is not available in the public domain. Therefore, prior to carrying out quantitative RT-PCR validation of cDNAs of interest identified via microarray analysis, we sequenced the Gambel's white-crowned sparrow cDNAs of interest. We used the publicly available cDNA sequence homologs of a related avian species, i.e. the zebra finch, as a basis to design RT-PCR primers (Table S8). The PCR amplicons were assessed via gel electrophoresis to ensure that only a single PCR product was generated. The PCR products were then sequenced. These empirically determined Gambel's white-crowned sparrow cDNA sequences were then used to design primers and probes for subsequent TaqMan-based RT-PCR analysis.

### Validation of Data Obtained with Microarrays Using Fluorogenic 5′-Nuclease-Based Assay and Quantitative RT-PCR

Fluorogenic 5′ nuclease-based assays were developed to quantitate the mRNA levels of specific genes. Briefly, reverse transcription was performed according to the SuperScript® III First-Strand Synthesis System (Invitrogen, Carlsbad, CA) manufacturer's protocol using the aRNA from previous microarray assays. We used 71 samples (HVC and RA from each bird used for the array portion of this study. There was not enough mRNA from RA in one animal) for rt-qPCR analysis. The antisense RNA samples were purified using the MEGAclear™ (Ambion, Austin, TX) prior to reverse transcription procedure. For gene expression measurements, 2 µL of cDNA were included in a PCR reaction (12 µL final volume) that also consisted of the appropriate forward (FP) and reverse (RP) primers, probes and TaqMan Gene Expression Master Mix (Applied Biosystems Inc., Foster City, CA). The PCR primers and the dual-labeled probes for the genes were designed using ABI Primer Express v.1.5 software (Applied Biosystems Inc., Foster City, CA). All oligo sequences are listed in Table S9.

Amplification and detection of PCR amplicons were performed with the ABI PRISM 7900 system (Applied Biosystems Inc., Foster City, CA) with the following PCR reaction profile: 1 cycle of 95°C for 10 min., 40 cycles of 95°C for 30 sec, and 60°C for 1 min. The 18S amplification plots derived from serial dilutions of an established reference sample were used to create a linear regression formula in order to calculate expression levels, and 18 s gene expression levels were utilized as an internal control to normalize the data.

Each measurement was done in triplicate wells on one plate, and each plate was run in duplicate or triplicate. Fold change between groups was calculated with the comparative C_T_ method. We note that qRT-PCR validation was done with the same biological specimens that were used for the microarray analysis. Thus our validation results do not provide any additional statistical rigor to the genes analyzed. Instead, they confirm that the pattern of expression holds across two different analytical platforms. Additional statistical rigor would depend upon validation using separate biological samples that could be measured, for instance, using semi-quantitative *in situ* hybridization.

### Hormone assay

Trunk blood was collected from each bird at sacrifice into a heparinized microhematocrit tube and stored on ice until centrifugation (within 1 h). We harvested the plasma and stored it at −20°C for subsequent steroid radioimmunoassay (RIA). To measure circulating T we followed the RIA protocol of Tramontin et al. (2001), using the Coat-a-Count total testosterone RIA kit (Diagnostic Products Corp., Los Angeles, CA). The minimum detectable plasma T concentration was 0.2 ng/ml.

## Supporting Information

Table S1 **All spots that varied by >1.5 fold in expression and had raw p-value<0.01 in HVC.** Numbers listed in table represent log fold change, and text in white color indicates a significant change. Key to the right illustrates color code for heat map for various levels of fold change. Note that some spots are assigned more than one Ensmbl ID.
(XLS)

Table S2 **All spots that varied by >1.5 fold in expression and had raw p-value<0.01 in RA.** Numbers listed in table represent log fold change, and text in white color indicates a significant change. Key to the right illustrates color code for heat map for various levels of fold change. Note that some spots are assigned more than one Ensmbl ID.
(XLS)

Table S3 **All spots that varied by >1.5 fold in expression and had raw p-value<0.01 in HVC realtive to LD+T Day 21 in animals shifted from breeding to nonbreeding conditions.** Numbers listed in table represent log fold change, and text in white color indicates a significant change. Key to the right illustrates color code for heat map for various levels of fold change. Note that some spots are assigned more than one Ensmbl ID.
(XLS)

Table S4 **All spots that varied by >1.5 fold in expression and had raw p-value<0.01 (white text) in RA realtive to LD+T Day 21 in animals shifted from breeding to nonbreeding conditions.** Numbers listed in table represent log fold change, and text in white color indicates a significant change. Key to the right illustrates color code for heat map for various levels of fold change. Note that some spots are assigned more than one Ensmbl ID.
(XLS)

Table S5 **Genes correlated with EGR1 expression.** (A) All spots in HVC that significantly co-varied with expression of EGR1 and had an ř2>0.5. (B) All spots in RA that significantly co-varied with expression of and had an ř2>0.5.
(XLSX)

Table S6 **All spots in HVC that significantly co-varied with expression in RA and had an ř2>0.5.** Spots that had an outlier which was greater than 3 times SD of the group are indicated. Spots that were also found to vary across breeding conditions in HVC and/or are indicated.
(XLSX)

Table S7 **Differences in gene expression between HVC and RA.** (A) All spots that were significantly upregulated in HVC in comparison to RA with a difference in expression of greater than 1.5 fold. (B) All spots that were significantly upregulated in RA in comparison to HVC with a difference in expression of greater than 1.5 fold.
(XLSX)

Table S8 **Primers for sequencing of the Gambel's white crowned sparrow cDNA.**
(XLSX)

Table S9 **Primers and probes for fluorescent 5′nuclease quantitative PCR assays.**
(XLSX)

## References

[1] Brenowitz EA, Zeigler HP, Marler P (2008). Plasticity of the adult avian song control system.. Neuroscience of birdsong.

[2] Nottebohm F (1981). A brain for all seasons: cyclical anatomical changes in song control nuclei of the canary brain.. Science.

[3] Nottebohm F (1987). Plasticity in adult avian central nervous system: possible relation between hormones, learning, and brain repair.. Handbook of physiology.

[4] Meitzen J, Perkel DJ, Brenowitz EA (2007). Seasonal changes in intrinsic electrophysiological activity of song control neurons in wild song sparrows.. J Comp Physiol A Neuroethol Sens Neural Behav Physiol.

[5] Catchpole C, Slater PJB (1995). Bird song: biological themes and variations.

[6] Zeigler HP, Marler P (2008). Neuroscience of birdsong.

[7] Reiner A, Perkel DJ, Bruce LL, Butler AB, Csillag A (2004). Revised nomenclature for avian telencephalon and some related brainstem nuclei.. J Comp Neurol.

[8] Replogle K, Arnold AP, Ball GF, Band M, Bensch S (2008). The Songbird Neurogenomics (SoNG) Initiative: community-based tools and strategies for study of brain gene function and evolution.. BMC Genomics.

[9] Wingfield JC, Farner DS (1978). The annual cycle of plasma irLH and steroid hormones in feral populations of the white-crowned sparrow, Zonotrichia leucophrys gambelii.. Biol Reprod.

[10] Smith GT, Brenowitz EA, Wingfield JC, Baptista LF (1995). Seasonal changes in song nuclei and song behavior in Gambel's white-crowned sparrows.. J Neurobiol.

[11] Thompson CK, Bentley GE, Brenowitz EA (2007). Rapid seasonal-like regression of the adult avian song control system.. Proc Natl Acad Sci U S A.

[12] Tramontin AD, Hartman VN, Brenowitz EA (2000). Breeding conditions induce rapid and sequential growth in adult avian song control circuits: a model of seasonal plasticity in the brain.. J Neurosci.

[13] Wu X, Watson M (2009). CORNA: testing gene lists for regulation by microRNAs.. Bioinformatics.

[14] Li M, Linseman DA, Allen MP, Meintzer MK, Wang X (2001). Myocyte enhancer factor 2A and 2D undergo phosphorylation and caspase-mediated degradation during apoptosis of rat cerebellar granule neurons.. J Neurosci.

[15] Butts BD, Linseman DA, Le SS, Laessig TA, Heidenreich KA (2003). Insulin-like growth factor-I suppresses degradation of the pro-survival transcription factor myocyte enhancer factor 2D (MEF2D) during neuronal apoptosis.. Horm Metab Res.

[16] Linseman DA, Bartley CM, Le SS, Laessig TA, Bouchard RJ (2003). Inactivation of the myocyte enhancer factor-2 repressor histone deacetylase-5 by endogenous Ca(2+)//calmodulin-dependent kinase II promotes depolarization-mediated cerebellar granule neuron survival.. J Biol Chem.

[17] Linseman DA, Cornejo BJ, Le SS, Meintzer MK, Laessig TA (2003). A myocyte enhancer factor 2D (MEF2D) kinase activated during neuronal apoptosis is a novel target inhibited by lithium.. J Neurochem.

[18] Butts BD, Hudson HR, Linseman DA, Le SS, Ryan KR (2005). Proteasome inhibition elicits a biphasic effect on neuronal apoptosis via differential regulation of pro-survival and pro-apoptotic transcription factors.. Mol Cell Neurosci.

[19] Tang X, Wang X, Gong X, Tong M, Park D (2005). Cyclin-dependent kinase 5 mediates neurotoxin-induced degradation of the transcription factor myocyte enhancer factor 2.. J Neurosci.

[20] Smith PD, Mount MP, Shree R, Callaghan S, Slack RS (2006). Calpain-regulated p35/cdk5 plays a central role in dopaminergic neuron death through modulation of the transcription factor myocyte enhancer factor 2.. J Neurosci.

[21] Wang X, She H, Mao Z (2009). Phosphorylation of neuronal survival factor MEF2D by glycogen synthase kinase 3beta in neuronal apoptosis.. J Biol Chem.

[22] Okamoto S, Li Z, Ju C, Scholzke MN, Mathews E (2002). Dominant-interfering forms of MEF2 generated by caspase cleavage contribute to NMDA-induced neuronal apoptosis.. Proc Natl Acad Sci U S A.

[23] Meitzen J, Weaver AL, Brenowitz EA, Perkel DJ (2009). Plastic and stable electrophysiological properties of adult avian forebrain song-control neurons across changing breeding conditions.. J Neurosci.

[24] Singh TD, Heinrich JE, Wissman AM, Brenowitz EA, Nordeen EJ (2003). Seasonal regulation of NMDA receptor NR2B mRNA in the adult canary song system.. J Neurobiol.

[25] Mello CV (2002). Mapping vocal communication pathways in birds with inducible gene expression.. J Comp Physiol A Neuroethol Sens Neural Behav Physiol.

[26] Anscombe FJ (1973). Graphs in Statistical Analysis.. The American Statistician.

[27] Goldman SA, Nottebohm F (1983). Neuronal production, migration, and differentiation in a vocal control nucleus of the adult female canary brain.. Proc Natl Acad Sci U S A.

[28] Thompson CK, Brenowitz EA (2008). Caspase inhibitor infusion protects an avian song control circuit from seasonal-like neurodegeneration.. J Neurosci.

[29] Song B, Meng F, Yan X, Guo J, Zhang G (2003). Cerebral ischemia immediately increases serine phosphorylation of the synaptic RAS-GTPase activating protein SynGAP by calcium/calmodulin-dependent protein kinase II alpha in hippocampus of rats.. Neurosci Lett.

[30] Huesmann GR, Clayton DF (2006). Dynamic Role of Postsynaptic Caspase-3 and BIRC4 in Zebra Finch Song-Response Habituation.. Neuron.

[31] Rothermundt M, Peters M, Prehn JH, Arolt V (2003). S100B in brain damage and neurodegeneration.. Microsc Res Tech.

[32] Lovell PV, Clayton DF, Replogle KL, Mello CV (2008). Birdsong “transcriptomics”: neurochemical specializations of the oscine song system.. PLoS One.

[33] Louissaint A, Rao S, Leventhal C, Goldman SA (2002). Coordinated interaction of neurogenesis and angiogenesis in the adult songbird brain.. Neuron.

[34] Jiang L, Zeng X, Yang H, Wang Z, Shen J (2008). Oral cancer overexpressed 1 (ORAOV1): a regulator for the cell growth and tumor angiogenesis in oral squamous cell carcinoma.. Int J Cancer.

[35] Vinci MC, Bellik L, Filippi S, Ledda F, Parenti A (2007). Trophic effects induced by alpha1D-adrenoceptors on endothelial cells are potentiated by hypoxia.. Am J Physiol Heart Circ Physiol.

[36] Spinazzi R, Albertin G, Nico B, Guidolin D, Di Liddo R (2006). Urotensin-II and its receptor (UT-R) are expressed in rat brain endothelial cells, and urotensin-II via UT-R stimulates angiogenesis in vivo and in vitro.. Int J Mol Med.

[37] Hartog TE, Dittrich F, Pieneman AW, Jansen RF, Frankl-Vilches C (2009). Brain-derived neurotrophic factor signaling in the HVC is required for testosterone-induced song of female canaries.. J Neurosci.

[38] Kim D-H, Lilliehook C, Roides B, Chen Z, Chang M (2008). Testosterone-Induced Matrix Metalloproteinase Activation Is a Checkpoint for Neuronal Addition to the Adult Songbird Brain 10.1523/JNEUROSCI.3674-07.2008.. J Neurosci.

[39] Kirn J, O'Loughlin B, Kasparian S, Nottebohm F (1994). Cell death and neuronal recruitment in the high vocal center of adult male canaries are temporally related to changes in song.. Proc Natl Acad Sci U S A.

[40] Tramontin AD, Brenowitz EA (1999). A field study of seasonal neuronal incorporation into the song control system of a songbird that lacks adult song learning.. J Neurobiol.

[41] Park SW, Yan YP, Satriotomo I, Vemuganti R, Dempsey RJ (2007). Substance P is a promoter of adult neural progenitor cell proliferation under normal and ischemic conditions.. J Neurosurg.

[42] Thompson CK, Brenowitz EA (2009). Neurogenesis in an adult avian song nucleus is reduced by decreasing caspase-mediated apoptosis.. J Neurosci.

[43] Hill KM, DeVoogd TJ (1991). Altered daylength affects dendritic structure in a song-related brain region in red-winged blackbirds.. Behav Neural Biol.

[44] Canady RA, Burd GD, DeVoogd TJ, Nottebohm F (1988). Effect of testosterone on input received by an identified neuron type of the canary song system: a Golgi/electron microscopy/degeneration study.. J Neurosci.

[45] Alvarez-Buylla A, Theelen M, Nottebohm F (1988). Birth of projection neurons in the higher vocal center of the canary forebrain before, during, and after song learning.. Proc Natl Acad Sci U S A.

[46] Scotto-Lomassese S, Rochefort C, Nshdejan A, Scharff C (2007). HVC interneurons are not renewed in adult male zebra finches.. Eur J Neurosci.

[47] Tramontin AD, Smith GT, Breuner CW, Brenowitz EA (1998). Seasonal plasticity and sexual dimorphism in the avian song control system: stereological measurement of neuron density and number.. J Comp Neurol.

[48] Meitzen J, Moore IT, Lent K, Brenowitz EA, Perkel DJ (2007). Steroid Hormones Act Transsynaptically within the Forebrain to Regulate Neuronal Phenotype and Song Stereotypy.. J Neurosci.

[49] Rasika S, Alvarez-Buylla A, Nottebohm F (1999). BDNF mediates the effects of testosterone on the survival of new neurons in an adult brain.. Neuron.

[50] Dittrich F, Feng Y, Metzdorf R, Gahr M (1999). Estrogen-inducible, sex-specific expression of brain-derived neurotrophic factor mRNA in a forebrain song control nucleus of the juvenile zebra finch.. Proc Natl Acad Sci USA.

[51] Li XC, Jarvis ED, Alvarez-Borda B, Lim DA, Nottebohm F (2000). A relationship between behavior, neurotrophin expression, and new neuron survival.. Proc Natl Acad Sci U S A.

[52] Chen X, Agate RJ, Itoh Y, Arnold AP (2005). Sexually dimorphic expression of trkB, a Z-linked gene, in early posthatch zebra finch brain.. Proc Natl Acad Sci U S A.

[53] Wissman AM, Brenowitz EA (2009). The role of neurotrophins in the seasonal-like growth of the avian song control system.. J Neurosci.

[54] Jiang J, McMurtry J, Niedzwiecki D, Goldman SA (1998). Insulin-like growth factor-1 is a radial cell-associated neurotrophin that promotes neuronal recruitment from the adult songbird edpendyma/subependyma.. J Neurobiol.

[55] Xie F, London SE, Southey BR, Annangudi SP, Amare A (2010). The zebra finch neuropeptidome: prediction, detection and expression.. BMC Biol.

[56] Li J, Zeng SJ, Zhang XW, Zuo MX (2006). The distribution of substance P and met-enkephalin in vocal control nuclei among oscine species and its relation to song complexity.. Behav Brain Res.

[57] Bottjer SW, Roselinsky H, Tran NB (1997). Sex differences in neuropeptide staining of song-control nuclei in zebra finch brains.. Brain Behav Evol.

[58] Mukai M, Replogle K, Drnevich J, Wang G, Wacker D (2009). Seasonal differences of gene expression profiles in song sparrow (Melospiza melodia) hypothalamus in relation to territorial aggression.. PLoS One.

[59] Newman AE, MacDougall-Shackleton SA, An YS, Kriengwatana B, Soma KK (2010). Corticosterone and dehydroepiandrosterone have opposing effects on adult neuroplasticity in the avian song control system.. J Comp Neurol.

[60] Katz A, Mirzatoni A, Zhen Y, Schlinger BA (2008). Sex differences in cell proliferation and glucocorticoid responsiveness in the zebra finch brain.. Eur J Neurosci.

[61] Seasholtz AF, Valverde RA, Denver RJ (2002). Corticotropin-releasing hormone-binding protein: biochemistry and function from fishes to mammals.. J Endocrinol.

[62] Sperry TS, Moore IT, Meddle SL, Benowitz-Fredericks ZM, Wingfield JC (2005). Increased sensitivity of the serotonergic system during the breeding season in free-living American tree sparrows.. Behav Brain Res.

[63] Sperry TS, Thompson CK, Wingfield JC (2003). Effects of acute treatment with 8-OH-DPAT and fluoxetine on aggressive behaviour in male song sparrows (Melospiza melodia morphna).. J Neuroendocrinol.

[64] Park KH, Meitzen J, Moore IT, Brenowitz EA, Perkel DJ (2005). Seasonal-like plasticity of spontaneous firing rate in a songbird pre-motor nucleus.. J Neurobiol.

[65] Hille B (2001). Ion channels of excitable membranes.

[66] Wade J, Arnold AP (2004). Sexual differentiation of the zebra finch song system.. Ann N Y Acad Sci.

[67] Jarvis ED, Nottebohm F (1997). Motor-driven gene expression.. Proc Natl Acad Sci U S A.

[68] Meitzen J, Thompson CK, Choi H, Perkel DJ, Brenowitz EA (2009). Time course of changes in Gambel's white-crowned sparrow song behavior following transitions in breeding condition.. Horm Behav.

[69] Kimpo RR, Doupe AJ (1997). FOS is induced by singing in distinct neuronal populations in a motor network.. Neuron.

[70] Velho TA, Pinaud R, Rodrigues PV, Mello CV (2005). Co-induction of activity-dependent genes in songbirds.. Eur J Neurosci.

[71] Pearen MA, Muscat GE (2010). Minireview: Nuclear hormone receptor 4A signaling: implications for metabolic disease.. Mol Endocrinol.

[72] Meitzen J, Thompson CK (2008). Seasonal-like growth and regression of the avian song control system: Neural and behavioral plasticity in adult male Gambel's white-crowned sparrows.. Gen Comp Endocrinol.

[73] Hidalgo A, Barami K, Iversen K, Goldman SA (1995). Estrogens and non-estrogenic ovarian influences combine to promote the recruitment and decrease the turnover of new neurons in the adult female canary brain.. J Neurobiol.

[74] Lombardino AJ, Hertel M, Li XC, Haripal B, Martin-Harris L (2006). Expression profiling of intermingled long-range projection neurons harvested by laser capture microdissection.. J Neurosci Methods.

[75] Key MD, Andres DA, Der CJ, Repasky GA (2006). Characterization of RERG: an estrogen-regulated tumor suppressor gene.. Methods Enzymol.

[76] Wood WE, Lovell PV, Mello CV, Perkel DJ (2011). Serotonin, via HTR2 Receptors, Excites Neurons in a Cortical-like Premotor Nucleus Necessary for Song Learning and Production.. J Neurosci.

[77] Camby I, Le Mercier M, Lefranc F, Kiss R (2006). Galectin-1: a small protein with major functions.. Glycobiology.

[78] Meitzen J, Thompson CK, Choi H, Perkel DJ, Brenowitz EA (2008). Time course of changes in Gambel's white-crowned sparrow song behavior following transitions in breeding condition.. Horm Behav.

[79] Tramontin AD, Wingfield JC, Brenowitz EA (2003). Androgens and estrogens induce seasonal-like growth of song nuclei in the adult songbird brain.. J Neurobiol.

[80] Schlinger BA, London SE (2006). Neurosteroids and the songbird model system.. J Exp Zoolog A Comp Exp Biol.

[81] Smyth GK (2004). Linear models and empirical bayes methods for assessing differential expression in microarray experiments.. Stat Appl Genet Mol Biol.

[82] Storey JD, Tibshirani R (2003). Statistical methods for identifying differentially expressed genes in DNA microarrays.. Methods Mol Biol.

[83] Harris JA, Iguchi F, Seidl AH, Lurie DI, Rubel EW (2008). Afferent deprivation elicits a transcriptional response associated with neuronal survival after a critical period in the mouse cochlear nucleus.. J Neurosci.

[84] Warren WC, Clayton DF, Ellegren H, Arnold AP, Hillier LW (2010). The genome of a songbird.. Nature.

